# Evaluation of carbon balance and carbohydrate reserves from forced (*Vitis vinifera L*.) cv. Tempranillo vines

**DOI:** 10.3389/fpls.2022.998910

**Published:** 2022-11-22

**Authors:** Jordi Oliver-Manera, Marina Anić, Omar García-Tejera, Joan Girona

**Affiliations:** ^1^ Efficient Use of Water in Agriculture Program, Institut of Agrifood Research and Technology (IRTA), Lleida, Spain; ^2^ Department of Viticulture and Enology, Faculty of Agriculture, University of Zagreb, Zagreb, Croatia; ^3^ Departamento de Agronomia, Instituto de Agricultura Sostenible – CSIC, Córdoba, Spain

**Keywords:** delayed ripening, forcing regrowth, photosynthesis, climate change, net carbon exchange, source:sink

## Abstract

Elevated temperatures during berry ripening have been shown to affect grape quality. The crop forcing technique (summer pruning that ‘force’ the vine to start a new cycle) has been shown to improve berry quality by delaying the harvest date. However, yield is typically reduced on forced vines, which is attributed to vine low carbon availability soon after forcing and likely incomplete inflorescence formation. The present study aims to estimate the carbon balance of forced vines and evaluate vine responses to changes in carbon patterns due to forcing. Three treatments were studied on Tempranillo cultivar: non-forced vines (Control), vines forced shortly after fruit set (CF_early_) and vines forced one month later at the beginning of bunch closure (CF_late_). Whole canopy net carbon exchange was modelled and validated using two whole canopy gas exchange chambers. In addition, non-structural carbohydrate reserves at budburst, forcing date and harvest, were analysed. Yield, yield components and vegetative growth were also evaluated. Harvest date was delayed by one and two months in the CF_early_ and CF_late_, respectively, which increased must acidity. However, yield was lower in the forced treatments compared to the Control (49% lower for CF_early_ and 82% for CF_late_). In the second year, at the time when CF_early_ and CF_late_ dormant buds were unlocked (forced budburst), forced vines had significantly lower non-structural carbohydrates than Control vines at budburst. Although the time elapsed from budburst to reach maximum net carbon exchange was longer for the Control treatment (80 days) than for the forced treatments (about 40 days), average daily net carbon exchange until harvest was comparable between Control (60.9 g CO_2_/vine/day) and CF_early_ (55.9 g CO_2_/vine/day), but not for CF_late_ (38.7 g CO_2_/vine/day). In addition, the time elapsed from budburst to harvest was shorter in forced treatments (about 124 days) than for the Control (172 days). As a result, the cumulative net carbon exchange until harvest was reduced by 35% (CF_early_) and 55% (CF_late_) in the forced treatments. However, no differences in carbon reserves at harvest were observed between treatments partly helped by the higher source:sink ratio observed in forced than Control vines.

## 1 Introduction

Temperatures in the Mediterranean region are expected to increase in the coming decades due to global warming (Allen et al., 2018). In the Ebro Valley (north-east of Spain), warming projections predict an advance of harvest dates of up to 18 days for the Chardonnay variety (Prats-Llinàs et al., 2017). Other studies predict an advance of 35 days for Tempranillo in the Duero Valley (Ramos et al., 2018). The effects of global warming are not limited to plant development. Berry and wine quality will be severely affected (Gutiérrez-Gamboa et al., 2021). At high temperatures, there is a decoupling between the accumulation of sugar and phenolic compounds in berries (Sadras and Moran, 2012), alcohol content may be higher and aroma, flavor, and acidity content lower in wine (Keller, 2010; Mira de Orduña, 2010).

To minimize the effects of global warming, it has been proposed to move the ripening period and harvest date to more suitable (cooler) conditions (Palliotti et al., 2014). Many different management techniques and strategies have been investigated including irrigation strategies, chemical treatments, late winter pruning, leaf removal, fruit thinning and severe trimming (Palliotti et al., 2014; Van Leeuwen and Destrac-Irvine, 2017; Santos et al., 2020; Gutiérrez-Gamboa et al., 2021). However, for most of these techniques, the delay in ripening would be limited to only a few (one or two) weeks with different effects on grape quality depending on the variety and the local climatic conditions. For instance, in a trimming study with Tempranillo cultivar conducted in a temperate Spanish region, a 5-day delay of veraison resulted in a decrease of the grape sugar-acid ratio (Santesteban et al., 2017). On the other hand, in a study carried out in a warmer region of Spain also with Tempranillo, the removal of all mature apical leaves caused a 10-day delay in the harvest date, but without any effect on sugar content and even reducing grape acidity at harvest in defoliated vines (Buesa et al., 2019). Forced regrowth (Gu et al., 2012) -better known as the crop forcing technique- can delay the berry ripening phase by up to two months (Martinez De Toda et al., 2019) resulting in a decrease of the sugar-acid ratio (Gu et al., 2012; Lavado et al., 2019; Martinez De Toda et al., 2019; Martínez-Moreno et al., 2019). The crop forcing technique involves heavy pruning from mid-spring to early summer, removing all leaves and clusters. A total of about 2-6 buds are left on the remaining shoots. The result is a break in dormancy in the remaining buds, forcing a new vegetative and reproductive cycle (Gu et al., 2012).

Promising results for improving berry quality at harvest, such as higher acidity and phenolic compound concentrations, have been obtained in Cabernet Sauvignon in California using the forcing technique (Gu et al., 2012). Some recent studies in Spain have reported similar improvements in fruit and wine quality in the Tempranillo cultivar. However, a significant reduction in yield and vine vigor has also been observed (Martinez De Toda et al., 2019; Martínez-Moreno et al., 2019; Lavado et al., 2019; Pou et al., 2019). This decrease in yield could be related to the phenological stage at which forced pruning is performed (Martinez De Toda et al., 2019). As a general rule, lower number of bunches per vine are observed the earlier the treatment, (Martinez De Toda et al., 2019; Martínez-Moreno et al., 2019) which suggests an incomplete inflorescence primordia formation at the time when vines are forced. It has also been suggested that the low carbohydrate availability at the time of forcing together with the environmental conditions of the forced vines, could explain the reduction in both yield (the reduced number of bunches per vine and berries per bunch) and vine vigour (Martínez-Moreno et al., 2019). Therefore, the two main limitations of the crop forcing technique are i) an incomplete inflorescence primordia formation before dormant bud unlocking and ii) the low carbohydrates availability caused by removing all leaves which can lead to a source limitation affecting yield for the current and the next year (Poni et al., 2020). An alternative crop forcing technique (named double cropping) in which neither the primary crop nor the leaves bellow the trimming point (node six) were removed, was validated on potted Pinot Noir vines by Poni et al. (2021). The dormant buds were successfully unlocked and, as a result, a forced crop was added to the primary crop in forced vines. In addition, the presence of new functional leaf area improved the whole vine carbon balance in forced vines compared to non-forced vines. The same technique was used in Tempranillo (Martínez De Toda, 2021), but the forced crop did not reach the total sugar concentration of the non-forced vines.

The use of whole-plant carbon balance simulation models is a powerful tool for analyzing the effects of canopy management on carbon dynamics, especially if the model is properly site validated. Poni et al. (2006) successfully adapted and validated the Charles-Edwards canopy photosynthesis model (Charles-Edwards, 1982) together with the Arrhenius equations for respiration to estimate the carbon balance of grapevines. This model is characterized by its simplicity and robustness when the vines are not under water stress. The net carbon exchange models, however, requires information about carbohydrate reserves to better interpret carbon dynamics (Holzapfel et al., 2010).

The aim of this study is to analyze the effect on carbon balance and carbon reserve dynamics of vines under the crop forcing technique to better interpret the effects on vine performance.

## 2 Materials and methods

### 2.1 Experimental site, plant material and experimental design

The experiment was conducted in 2018 and 2019 in a commercial vineyard in Lleida (41.65°N, 0.52°E; 320 m.a.s.l.) on Tempranillo vines grafted on R110 rootstock and planted in 2013. The rows were north-south oriented (31.6°N-E) with 1.65 m between vines and 2.5 m between rows. Vines were trained with double cordons and had a vertically-positioned canopy. The criterion for winter pruning was to leave about 12 spurs on each vine and two buds per spur. Vines were drip irrigated with 2.3 L/h emitters spaced at 0.6 m intervals. Irrigation scheduling was calculated using the water balance approach (Allen et al., 1998) and based on a stem water potential threshold of -0.8 MPa, as proposed by Marsal et al. (2008) for non-stressed vines.

Weather data were collected from a weather station located 6.8 km from the plot. The weather station forms part of the regional weather service of Catalonia. In 2019, phenology was assessed weekly according to the modified E-L system (Coombe, 1995). Growing degree days (GDD) were calculated with 10°C as the baseline temperature.

Three treatments were studied: unforced vines grown conventionally according to winery criteria (Control), early forcing (CF_early_) and late forcing (CF_late_). In 2019, in Control vines, a standard removal of excessive/basal lateral shoots was undertaken on July 2, together to the removal of the bunch zone leaves (bellow nodes 3-6) to allow exposure of the bunch to the sun, while a mechanical hedging and lateral trimming was executed on July 10, and a fruit thinning to retain about 20 bunches per vine was operated on 19 July. Forcing treatments were applied shortly after fruit set (E-L 27) for CF_early_ and at the beginning of bunch closure (E-L 32) for CF_late_. The exact dates were June 14 and June 3 for CF_early_ and July 13 and July 1 for CF_late_, for 2018 and 2019, respectively. Crop forcing pruning was performed mechanically with a pre-pruner (Pellenc DISCO, Pellenc SAS, Pertuis, France) attached to a tractor, retaining 6-8 buds per shoot, and manually removing the remaining leaves and bunches. It should be taken into account that the phenological shift due to the crop forcing technique required adaptation of pest and disease control, as well as irrigation system and irrigation scheduling to each treatment. Therefore, the experimental design was designed to allow viticulture personnel to manage each treatment differently. Based on a high resolution NDVI map performed in 2016 of the same field and described in Bellvert et al. (2020), in an area as much homogeneous as possible, three parallel and adjacent plots were established with four rows and 12 vines per row (**Supplementary Figure 1** in **Supplementary Material**). Each plot was randomly assigned a treatment. The most likely gradient of vigor was observed following the direction of the vine rows. Then, the central 16 vines of each plot were grouped into four replicates of four vines each, according to the orientation of the plot. The maximum distance between two vines of each replicate of different treatments was 22.5 m (between the Control and the CF_early_). Before any treatment was applied, trunk diameter perpendicular to the row at a 0.5 height was measured in each vine of each plot and statistical analysis were performed (**Supplementary Table 1** in the **Supplementary Material**). Trunk diameters were 38.0 cm (Control), 41.2 cm (CF_early_) and 40.9 cm (CF_late_). No statistical differences were observed between treatments (*P* = 0.28) but between replicates (*P* = 0.01). The edge vines of each plot were used as buffers to avoid the influence of the neighboring treatment. All the vine measurements are described in the next sections. However, due to the large amount of data from the experiment, all measured, and some estimated parameters are summarized in **Supplementary Table 2**.

### 2.2 Yield, yield components, grape quality, and carry-over effects

The optimal harvest date was set at a total soluble solids content (TSS) between 22.5 and 23.5 °Brix for all treatments. Thus, from one month after veraison until harvest, a sample of berries was collected approximately every three days to extract the juice and measure TSS with a refractometer (Pallette, PR-32α, ATAGO Co., LTD., Tokyo, Japan) to ensure the optimal harvest date. At harvest, yield, number of bunches per vine and bunch weight were determined. A sub-sample of 50 berries per replication was weighed (berry weight). The number of berries per bunch and per vine was then estimated. All samples were weighed shortly after collection and dried at 65°C to constant weight to determine the dry weight. Berry juice was extracted from one sample of each replication and TSS was determined. The same juice was used to measure pH using a pHmeter (Crison PLG-22, HACH LANGE, SLU, Barcelona, Spain) and titratable acidity (TA). To measure TA (g/L tartaric acid) of the must, 10 mL of filtered juice was diluted with 10 mL of distilled water and titrated with a 0.1 N NaOH solution to a final pH of 8.2. In 2019, bunch compactness was estimated from the ratio between bunch and rachis weight of a subsample of 10 bunches per treatment at harvest. Before performing the forcing in 2019, the number of bunches per shoot on one vine per replicate was counted to evaluate carry-over effects from the 2018 season.

### 2.3 Vegetative growth, light interception, and biomass

In 2018, the winter pruning of 10 vines per treatment was collected and weighed, and the number of shoots of one vine per replicate was counted.

In 2019, one vine per replicate was selected (four vines per treatment) to measure biomass (leaves, shoots, and fruit), leaf area (LA), trunk cross-sectional area (TCSA) and the fraction of intercepted photosynthetically active radiation (FIPAR). On forcing dates (June 3 for CF_early_ and July 1 for CF_late_) total biomass removed by forcing pruning was collected and weighed. Shoots and bunches were also counted. On each forcing date, four vines located outside the experiment managed as the Control treatment were forced. Biomass was then measured, and shoots and bunches were counted. In addition, total biomass removed by the vineyard management actions described in section 2.1 for the Control treatment was recorded. In the forced vines, no management actions were performed other than forcing except for light defoliation of CF_early_ vines in September which was also weighed. At the end of the season, the four selected vines per treatment were bagged and their leaves and shoots collected and counted. The collected biomass was dried at 65°C until constant weight and then weighed.

Every three weeks, LA was determined on four representative fruiting shoots per vine using the Lopes and Pinto procedure (Lopes and Pinto, 2005). In this procedure, total LA is estimated by multiplying the leaf area of individual shoots by the number of shoots per vine. The leaf area of the individual shoot was calculated as the mean of the leaf with the largest and smallest area on the shoot multiplied by the number of leaves per shoot. Individual leaf area was then estimated from leaf central vein (CV) measurements using a linear regression between the two parameters (L*A* = 21.531*CV*– 93.98; *R* ^2 =^ 0.89). To obtain the regression, 150 leaves per treatment were sampled from July to October. For each leaf, the central vein was measured with a tape-measure and individual leaf area was measured with the Li3000 (Li-3000, Li-Cor, Inc., Lincoln, NE, USA). Only leaves in which the central vein was longer than 4.5 cm were considered.

Trunk diameter was calculated as the average of two diameter measurements per vine taken with a digital caliper (Absolute Digimatic Caliper, Mitutoyo Corp., Aurora, IL, USA) parallel and perpendicular to the row at a height of 50 cm above the ground. Then, the increase in trunk cross-sectional area (ΔTCSA) was calculated as the difference in TCSA between the beginning and end of the season.

The fraction of photosynthetically active radiation intercepted by the vines (FIPAR) was measured every three weeks from May to the end of September. This was carried out between 11:00 and 12:00 (GMT) using an Accupar LP-80 ceptometer (Meter group, Inc. USA). For each vine, one measure was taken above the canopy (I_above_) and 12 measurements were taken below the canopy (I_below_). Measurements below the canopy were taken parallel to the row and 0.5m apart to cover the entire ground allocated per vine. The FIPAR was calculated as follows (Equation 1):


(1) $$\text{FIPAR}=\;1-\frac{\sum^{}I_{below}/12}{I_{above}}$$

These punctual FIPAR measurements were used as input for estimating daily FIPAR using a model based on the specific site and plant characteristics (canopy height and width) measured on the same dates (Oyarzun et al., 2007).

The winter pruning weight of 10 vines per treatment was also measured.

### 2.4 Plant water status, leaf photosynthesis, and quantum yield

Physiological measurements were made during the 2019 season from May to October. Stem water potential (Ψ_s_) was measured every 15 days from May to October following the Shackel et al. (1997) methodology. On four vines in each treatment (one per replicate), a shaded leaf located near the trunk was bagged in an aluminum bag 30 min before the measurement. Measurements were carried out between 11:30 and 12:30 (GMT), using a pressure chamber (Model 3005, Soil moisture, Corp. Sta. Barbara, CA, USA).

Stomatal conductance (g_s_) and leaf net photosynthesis (Pn) were determined using an LCA-4 portable open gas exchange system (ADC, Hoddesdon, UK). Measurements were performed on two fully developed, sunlit leaves (PAR > 1200 μmol/s/m^2^) per vine, located at mid-height of the canopy. Measurements were made monthly at between 11:00 and 12:30 (GMT) on the same four vines per treatment in which biometric measurements were realized. Therefore, a total of 8 measurements per treatment were performed on each measurement date.

Quantum yield (α) was measured, approximately once a month, on one fully expanded and sunlit leaf from three vines per treatment. A Li-6400 infrared gas analyser system (Li-6400, Li-Cor, Inc., Lincoln, NE, USA), equipped with a 6400-40 leaf chamber fluorometer (10% blue light and 90% red light), was used to establish the photosynthetic response to photon flux density (Pn/I curves) within a photon flux density range of 50, 100 and 200 μmol/s/m^2^. Leaf temperature was set at 25 ± 2°C, except in October, when a leaf temperature of 20 ± 2 °C was considered more appropriate. The reference CO_2_ concentration was set at 400 ppm. Measurements were performed from 7:00 to 11:00 (GTM) to avoid a relative humidity lower than 40% as proposed by Escalona et al. (1999).

### 2.5 Canopy photosynthesis model

A simple variant of the Charles-Edwards (1982) model was used to estimate daily canopy photosynthesis during the 2019 growing season (Equation 2) (Poni et al., 2006). Data obtained from the same four vines per treatment where biomass, leaf area and FIPAR were monitored were used to run the model. This model is based on the Big Leaf approach and requires light and plant characteristics as inputs:


(2) $${\text{Pn}}_{\text{canopy}}=\;\frac{\propto \text{ S h dailyFIPAR Pn}}{\propto \text{k S}+\text{h Pn}}\text{ G}$$

where Pn_canopy_ is the net photosynthesis of all the canopy leaves (g CO_2_/vine/day); α is the quantum yield (μg CO_2_/J); S is the total daily integral of PAR radiation on the horizontal plane (MJ/m^2^/day); h is daylength (s); Pn is leaf photosynthesis (mg CO_2_/m^2^/s); k is extinction coefficient (dimensionless); and G is vine spacing (m^2^/vine). We assumed that 48% of the total incident radiation was in the PAR range (Tsubo and Walker (2005)).

k is based on Russell et al. (1989) (Equation 3):


(3) k= ln(IaboveIbelow)LAI 

where I_above_ is the incident radiation above the canopy and I_below_ is the radiation measured bellow the canopy described in section 2.3, and LAI is the leaf area index, calculated as leaf area per ground area (m^2^/m^2^).

Parameters that fell between two different measured parameters were estimated linearly to allow the model to run continuously.

### 2.6 Net carbon exchange model

Using the same four vines per treatment for which Pn_canopy_ was calculated, daily net carbon exchange was estimated as follows (Equation 4):


(4) $${\text{NCE}}_{\text{m}}=\sum_{\text{sunrise}}^{\text{sunset}}{\text{Pn}}_{\text{canopy}}-\sum_{\text{sunset}}^{\text{sunrise}}{\text{R}}_{\text{leaf}}-\sum_{0}^{24}{\text{R}}_{\text{shoot}}-\sum_{0}^{24}{\text{R}}_{\text{fruit}}-\sum_{0}^{24}{\text{R}}_{\text{trunk}}$$

where NCE_m_ is the modelled daily net carbon exchange (g CO_2_/day), Pn_canopy_ is calculated canopy net photosynthesis (g CO_2_/day) from sunrise to sunset; and R is respiration of aerial organs (g CO_2_/day). Because leaf respiration during the day was already included in Pn_canopy_, only nocturnal leaf respiration was estimated. Respiration was calculated according to Equation 5:


. (5) $$\text{R}={\text{ q}}_{\text{m}}{\text{ X Q}}_{10}^{\left(\frac{\text{T}}{10}\right)}$$

where q_m_ is the maintenance coefficient, X is the organ size parameter (g DW or m^2^), Q_10_ is the temperature coefficient and T is the air temperature (°C). Both q_m_ and Q_10_ were taken from the literature (Palliotti et al., 2005; Poni et al., 2006; Escalona et al., 2012 and Hernández-Montes et al., 2020) (see **Supplementary Table 3** in the **Supplementary Material**). It was assumed that respiration coefficients did not change because of the treatment. Respiration coefficients that fell between two different phenological stages were estimated linearly. The organ size parameters (X) were LA, trunk area, fruit dry weight (DW), and shoot DW. Trunk area was calculated as the product of the perimeter of the trunk by the trunk height. From pea size of berry development (E-L31) to harvest, 2 berries were collected from ten vines per treatment at approximately 15-day intervals, dried at 65°C, and weighed. Fruit DW was then estimated by multiplying the dry weight of each berry by the final number of berries at harvest. Shoot DW was estimated from shoot length of the same shoots on which LA was measured multiplied by the number of shoots per vine. The conversion from shoot length to shoot dry weight was calculated using a linear regression between shoot density (SD) (g DW/cm) and GDD (*SD* = 0.00005 *GDD* + 0.0394; *R* ^2^ = 0.95). To obtain the regression, 16 shoots per treatment were measured, dried at 65°C, and weighed on each forcing pruning date (June 3 and July 1) and at the end of the season (November 18). The organ size parameters that fell between measurement dates were estimated linearly using GDD as the physiological time scale.

The modelled net carbon exchange was normalised relative to the total LA of each vine (NCE/LA) and expressed as g CO_2_/m^2^ leaf area.

### 2.7 Canopy net carbon exchange 7model validation

To validate the model, two open-top gas exchange chambers were constructed which were similar to those described by Corelli-Grappadelli and Magnanini (1993) (see **Supplementary Figure 2**). The chamber volume was approximately 5 m^3^ and they were made of Mylar^R^ plastic. Air was introduced into the chamber from a height of 3.5 m *via* a 20 cm diameter aluminum tube. A 186.5 W centrifugal fan (Casals Ventilación Industrial IND, S.L., Girona, Spain) was installed in each chamber to blow the air into the chamber through a 19 cm diameter PVC pipe. Once the air was in the chamber, it was distributed through a perforated aluminum tube around the base of the canopy. Air mixing inside the chamber was enhanced by two 12V cpu fans (F12 PWM PST, Arctic GmbH), positioned directly above the canopy. The air velocity (m/s) at the center of the inlet tube was measured using an air velocity transmitter (Dwyer Series 641, USA) and the flow rate (F_v_), in m^3^/s, was calculated as follows (Equation 6):


(6) Fv  = air velocity · pipe area.

Air flow was adjusted to approximately 12 m^3^/min using a REG-5 fan speed controller (Casals Ventilación Industrial IND, S.L., Girona, Spain). Temperature and relative humidity inside and outside the chamber were monitored using a Vaisala HMP110 sensor (Vaisala Corporation, Helsinki, Finland). Global solar radiation was measured inside and outside the chamber using pyranometers (Apogee SP-110, Apogee Instruments, Inc., North Logan, USA). CO_2_ concentrations at the inlet (CO_2 ref_) and outlet (CO_2 an_) were measured using an infrared gas analyzer (Li-820, Li-Cor, Inc., Lincoln, NE, USA). A handmade system of valves, micropumps, and tubes controlled by an SDM-CD16AC relay controller (Campbell Scientific, Inc., N Logan, USA) was used to pump the air from the inlet and outlet of the chamber to the gas analyzer device. The delay between each reference and corresponding analysis measurement was 40 s. Net carbon exchange in the chamber was calculated as follows (Equation 7):


(7) $${\text{NCE}}_{\text{ch}}={\text{F}}_{\text{m}}\left({\text{CO}}_{2\text{ ref}}-\text{CO}2_{2\text{ an}}\right)\;$$

where NCE_ch_ is the net carbon exchange in the chamber (μmol CO_2_/s/vine), and F_m_ is the air flow (mol/s) which was calculated as follows (Equation 8):


(8) $${\text{F}}_{\text{m}}={\text{F}}_{\text{v}}\frac{1000}{22.41}\frac{273.15}{\left(\text{T}+273.15\right)}$$

where T is the air temperature in Celsius.

The recording frequency was 20s using a CR1000 datalogger equipped with two AM16-32B multiplexers (Campbell Scientific, Inc., N Logan, USA).

The model was validated by comparing the sum of NCE_m_ with the sum of NCE_ch_ (g CO_2_/vine). Measurements were taken from May to the end of September on 8 different days from approximately 6:00 to 17:00 (GMT). On each day, a Control and a CF vine were measured simultaneously. The coefficient of determination (R^2^), root mean square error (RMSE) and the Nash-Sutcliffe Efficiency (NSE) coefficient were used to validate the model.

### 2.8 Carbohydrate reserves

Carbohydrate reserves were evaluated at the beginning of the 2019 season shortly after budbreak (April 2), at forcing pruning dates (June 3 for CF_early_ and July 1 for CF_late_) and at harvest (September 16 for Control, October 16 for CF_early_ and November 11 for CF_late_). On each sampling date, one trunk and one root sample (with a diameter between 5-10 mm) were collected from three vines per treatment. Because the sampling procedures were destructive, samples were collected from vines at the edges to avoid affecting vines used for other measurements. Trunk samples were collected with a corer inserted 10 mm from mid trunk heigh for each vine after removing the bark. Two holes per vine were made to ensure that there were sufficient sample for analysis. All samples were stored in a portable refrigerator and quickly transported to the laboratory. At the laboratory, samples were microwaved at 600W for 90 s to inactivate enzymes, as described by Landhäusser et al. (2018), and shortly thereafter oven dried at 65°C to constant weight. To determine soluble sugars concentration, 50 mg of the ground sample was used following a modification of the phenol-sulfuric acid colorimetric method (Buysse and Merckx, 1993). The solid residue of the process was used to determine the starch concentration by using amyloglucosidase to hydrolyze the starch. The starch concentration was determined by spectrophotometry at 490 nm as glucose. The total concentration of non-structural carbohydrates (%DW) in the trunk (TNSC) and root (RNSC) was calculated as the sum of soluble sugars and starch.

### 2.9 Statistical analysis

A univariate analysis of variance (ANOVA) was performed to reveal differences between treatments (*P<* 0.05). The normal distribution of experimental errors was assessed with Shapiro-Wilk test. Homogeneity of error variances was assessed with Levene’s test (*P<* 0.05). Differences between means were determined using the Tukey test. All statistical analyses were performed with JMP14 software (SAS Institute Inc., Cary, NC, 1989-2021).

## 3 Results

### 3.1 Weather and phenological data

**Figure 1:**
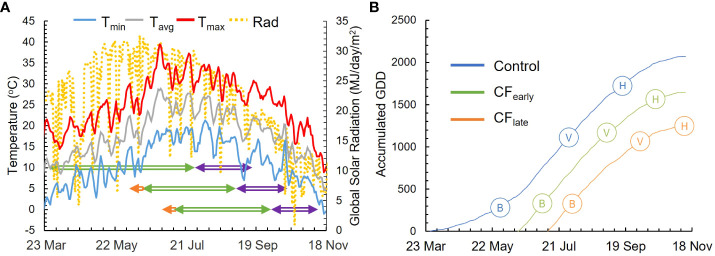
Weather and phenological data for season 2019. Daily maximum (red line), mean (grey line), and minimum (blue line) temperatures and daily global solar radiation (dotted yellow line) are represented. Phenological periods represented are from budburst to veraison (green arrowed line) and from veraison to harvest (purple arrowed line). Period from forcing treatment to budburst is also represented (orange arrowed line) **(A)**. The seasonal cumulative GDD for 2019 season for Control (blue line), CF_early_ (green line) and CF_late_ (orange line) **(B)**. Full bloom (B), veraison (V) and harvest (H) are also represented.

In 2018, vines were harvested on September 21 (Control), October 26 (CF_early_) and November 27 (CF_late_). In 2019, budburst (E-L 4) was observed on March 26 (Control), on June 14 (CF_early_), 11 days after forcing, and on July 11 (CF_late_), 10 days after forcing (**Figures 1A, B**). Veraison (E-L 35) of 50% of berries was observed on July 26 (Control), September 2 (CF_early_), and October 2 (CF_late_) while harvest dates were on September 16 (Control), October 16 (CF_early_), and November 11 (CF_late_). The period from budburst to veraison lasted 127 days (1112 GDD) for Control, 81 days (1179 GDD) for CF_early_ and 84 days (1082 GDD) for CF_late_ treatments. Average temperatures from budburst to veraison were 18.3, 24.3 and 22.5°C for the Control, CF_early_ and CF_late_ vines, respectively (**Figure 1B**). Average ripening temperatures were lower in the forcing treatments (22.8, 19 and 14°C for the Control, CF_early_ and CF_late_ vines, respectively). Mean daily global solar radiation was similar between treatments before veraison but was reduced by 18% and 48% for the CF_early_ and the CF_late_ treatments respectively, during the ripening period compared to Control (**Figure 1A**).

### 3.2 Vine performance and grape quality

In 2018, vines were severely affected by downy mildew in May and early June which affected some inflorescences and fruits. Therefore, yield was negatively affected in the Control treatment although individual bunch weight at harvest was still higher than in the forced treatments (**Table 1**). Because downy mildew attack occurred before forcing, yields of the forced treatments were not affected. Forced vines reduced pruning weight by about 40% (**Table 1**). As a result, the highest Ravaz index was observed in CF_early_ vines because yield was the same as the Control but with lower vigour.

**Table 1:** Effects of crop forcing on yield components, grape quality, and vine vigour in 2018 and 2019.

Season	Treatment	TSS °Brix	TA (g/L)	pH	Bunches per vine (No)	Shoots per vine (No)	Bunches per shoot (No)	Bunch weight (g)	Berryweight (g)	Berriesper bunch (No)	Berriesper vine (No)	Yield (kg/vine)	Pruning weight (kg/vine)	Ravaz Index (kg/kg)
2018	Control	22.4 ± 0.1 b	5.8 ± 0.1 c	3.6 a	17 ± 2 b	22 ± 1 b	0.8 ± 0.1 b	200 ± 9 a	2.39 ± 0.09 a	83 ± 4 a	1455 ± 176b	3.5 ± 0.4a	1.46 ± 0.10a	2.4 ± 0.3b
	CF_early_	23.8 ± 0.1 a	8.4 ± 0.3 b	3.3 b	33 ± 4 a	28 ± 2 a	1.3 ± 0.1 a	119 ± 6 b	1.62 ± 0.10 c	74 ± 4 b	2435 ± 316a	3.9 ± 0.5a	0.83 ± 0.07b	4.4 ± 0.6a
	CF_late_	21.6 ± 0.0 c	12.5 ± 0.5 a	3.1 c	5 ± 0 c	22 ± 1 b	0.2 ± 0.0 c	34 ± 10 c	1.23 ± 0.03 b	30 ± 8 c	128 ± 21c	0.2 ± 0.1b	0.89 ± 0.06b	0.2 ± 0.0c
	Significance	*	*	*	*	*	*	*	*	*	*	*	*	*
2019	Control	23.4 ± 0.1	4.7 ± 0.0 c	3.3 a	23 ± 1 b	27 ± 1 b	0.9 ± 0.0 a	387 ± 31 a	2.56 ± 0.05 a	141 ± 14 a	3364 ± 199a	8.7 ± 0.5a	0.99 ± 0.14a	8.8 ± 0.5a
	CF_early_	23.4 ± 0.1	6.9 ± 0.2 b	3.3 a	19 ± 2 b	38 ± 4 ab	0.5 ± 0.0 b	118 ± 6 b	2.16 ± 0.05 b	55 ± 3 b	1095 ± 154b	2.4 ± 0.3b	0.99 ± 0.21a	2.4 ± 0.4c
	CF_late_	23.7 ± 0.2	10.2 ± 0.3 a	3.1 b	36 ± 3 a	42 ± 6 a	0.9 ± 0.1 a	58 ± 4 b	1.51 ± 0.02 c	38 ± 3 b	1307 ± 101b	2.0 ± 0.2b	0.42 ± 0.10b	4.8 ± 0.4b
	Significance	ns	*	*	*	*	*	*(*)	*	*(*)	*	*	*	*

Treatment effects were analysed using ANOVA and the means were separated with the Tukey test. Means followed by different letters are different at P< 0.05. Significance levels in brackets correspond to differences only analysing forced treatments through t-test. ns, not significant. *=significant. The grape quality parameter data are the mean of 4 samples (one per each replication) per treatment ± standard error. Yield, bunches per vine, bunch weight, berries per bunch and berries per vine at harvest data are the means of 16 vines per treatment ± standard error. Berry weight is the mean of 50 berries from each of four replications per treatment. Shoots per vine are the means of four vines (one per each replication) per treatment ± standard error. Pruning weight data are the means of 10 vines per treatment ± standard error.

In 2019, both forced treatments reduced yield and yield components compared to the Control, except for the number of bunches per shoot, which was reduced in CF_early_ but not in CF_late_ (**Table 1**). The CF_early_ exhibited a higher bunch weight due to a higher number of berries per bunch and higher berry weight compared to CF_late_. Bunch compactness was not affected by the forcing (**Table 2**). Lower pruning weight was observed only in the CF_late_ treatment (**Table 1**). Control vines had a higher Ravaz index (**Table 1**) and a lower LA/fruit ratio than forced vines (**Table 2**). We did not observe any differences in trunk growth between treatments (**Table 2**).

**Table 2:** Effects of crop forcing on number of bunches per shoot before forcing (before fruit thinning and shoot removal for the Control treatment), bunch compactness at harvest, LA/fruit ratio and TSCA in 2019.

Parameter	Control	CF_early_	CF_late_	Significance level
Number of bunches per shoot before forcing (No)	1.7 ± 0.2a	0.8 ± 0.0b	1.5 ± 0.1a	*
Bunch compactness (g/g)	26.8 ± 0.7	29.6 ± 1.3	30.7 ± 1.0	ns
LA/fruit (cm^2^/g)	7.4 ± 0.7c	35.3 ± 2.8a	22.7 ± 2.5b	*
ΔTCSA (cm^2^/vine)	3.3 ± 0.7	3.1 ± 0.7	2.7 ± 0.2	ns

Treatment effects were analysed using ANOVA and the means were separated with the Tukey test. Means followed by different letters are different at P< 0.05. ns, not significant. *=significant. Bunch compactness is the mean of 10 bunches ± standard error. LA/fruit ratio and ΔTCSA data are the means of four vines (one per replication) per treatment ± standard error.

Yield was reduced by 39% in CF_early_ but increased 10-fold in CF_late_ in 2019 when compared to 2018. In contrast, pruning weight increased 12.5% in CF_early_ but decreased by 50% in CF_late_ in 2019 when compared to 2018.

Regarding the berry quality traits, differences between treatments were observed in 2018 in TSS because sugar accumulation stopped in November 15 in CF_late_ treatment (data not shown), but not in 2019 (**Table 1**). An increase in TA was observed in the forced treatments. The later the harvest date, the greater the TA. Except in 2019 when the same pH was observed for the Control and the CF_early_ treatments, the must pH was generally lower in forced treatments.

### 3.3 Leaf area and fraction of intercepted radiation

**Figure 2:**
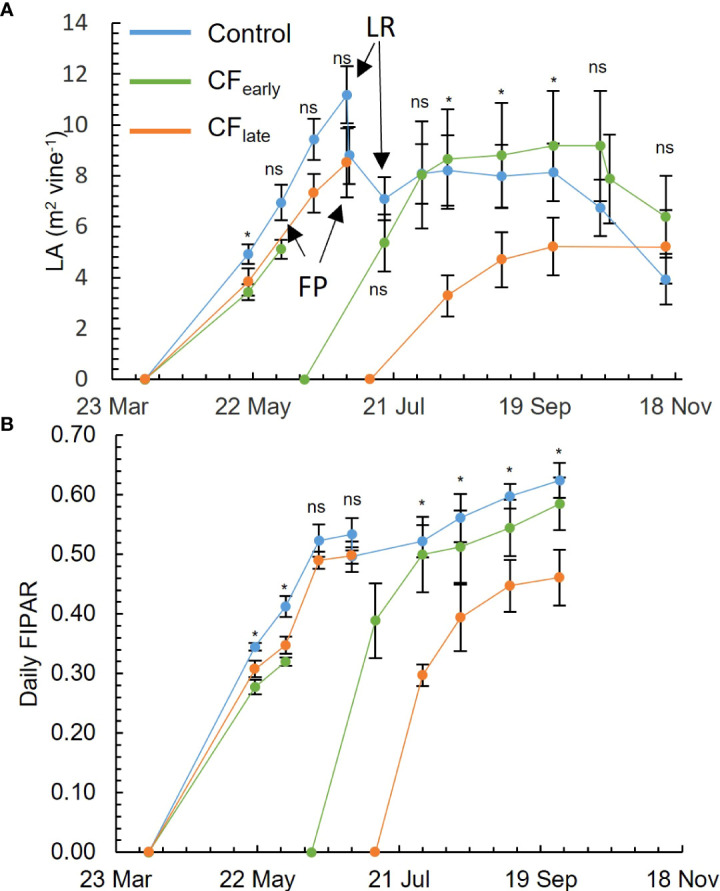
Seasonal evolution for 2019 of leaf area (LA). FP, forcing pruning; LR, leaf and shoot removal, only affecting control treatment **(A)**. Daily fraction of intercepted radiation (FIPAR) evolution for each treatment modelled according to the Oyarzun et al. (2007) model **(B)**. Blue, green, and orange lines correspond to Control, CF_early_ and CF_late_ treatments, respectively. Each plot is the average of four vines for each treatment. Bars mean standard deviation. Treatment effects were analysed using ANOVA at P< 0.05. ns, not significant; *=significant.

Before forcing in 2019, differences in LA were observed between the Control and CF_early_ in the measurement taken on May 20, but not after (**Figure 2A**). On the other hand, differences in FIPAR were observed between the Control and both forced treatments until the measurement taken on June 3 (**Figure 2B**). After forcing in 2019, CF_early_ vines reached LA and FIPAR values comparable to Control within approximately one and a half months after budburst (**Figures 2A, B**). CF_late_ vines failed to reach maximum levels of LA and FIPAR comparable to the other treatments (**Figures 2A, B**). However, compared to Control, the reduction of LA was higher than the reduction of FIPAR (36% and 26% respectively), which may be related to a sparser canopy (visual observation) in CF_late_ vines.

### 3.4 Biomass partitioning

Although dry matter partitioned to fruit and shoots differed between 2018 and 2019, the sum of both was maintained in the forced treatments (**Table 3**). In 2019, biomass dry matter was higher in the Control than for the forced treatments (**Table 4**). Forcing reduced biomass by 20% and 54% in the CF_early_ and CF_late_ treatments, respectively. After forcing, total biomass was 41% lower in CF_late_ than in CF_early_. The relative proportion of biomass partitioned among vegetative organs (leaves + shoots) after forcing was 62.2% in CF_early_ and 48% in CF_late_, compared to 31.5% in Control vines. The CF_late_ vines invested a lower proportion of DM in the shoots, but the same in the leaves than the CF_early_ vines.

**Table 3:** Effects of crop forcing on dry matter partitioned to fruit and shoots between years 2018 and 2019.

	Year	Control	CF_early_	CF_late_
Fruit DM (g)	2018	808 ± 65	1008 ± 16	50 ±7
	2019	2541 ± 249	686 ± 170	551 ± 72
Significance		*	ns	*
Shoot DM (g)	2018	786 ± 56	450 ± 16	486 ± 19
	2019	532 ± 75	536 ± 114	224 ± 53
Significance		*	ns	*
Fruit + Shoot DM (g)	2018	1593 ± 107	1458 ± 25	536 ± 24
	2019	3073 ± 296	1222 ± 282	776 ± 115
Significance		*	ns	ns

Treatment effects were analysed using t-test at P< 0.05. ns, not significant. *=significant. Data are the means of four replicates ± standard error.

**Table 4:** Biomass removed and distribution on forcing dates and at the end of the season in year 2019.

Date	Treatment	B_t_ (g DW/vine)	%fruit	%leaves	%shoots
3 June	Control	714 ± 79	7.2 ± 0.9	69.6 ± 2.6	23.2 ± 1.7
	CF_early_	505 ± 46	2.4 ± 0.3	70.3 ± 3.2	27.3 ± 3.5
Significance level		*	*	ns	ns
1 July	Control	1737 ± 168	33.2 ± 3.3	47.2 ± 1.7	19.6 ± 1.8
	CF_late_	1318 ± 255	34.5 ± 1.0	49.0 ± 2.0	16.5 ± 1.1
Significance level		ns	ns	ns	ns
Whole season	Control	4284 ± 478 a	68.5 ± 1.8 a	17.9 ± 0.9 b	13.6 ± 1.0b
	CF_early_	2453 ± 548 b	29.9 ± 3.1 b	42.0 ± 2.1 a	28.1 ± 1.3a
	CF_late_	2844 ± 623 b	42.3 ± 3.0 b	39.6 ± 2.7 a	18.1 ± 1.0b
Significance level		*	*	*	*
Forced season	CF_early_	1947 ± 507 b	37.8 ± 2.6 c	33.1 ± 1.6 a	29.1 ± 2.6a
	CF_late_	1156 ± 293 c	52.0 ± 5.1 b	29.8 ± 3.7 a	18.2 ± 1.8b
Significance level		*	ns	ns	*

Treatment effects were analysed using ANOVA and the means were separated with the Tukey test. Means followed by different letters are different at P< 0.05. ns, not significant. *=significant. Data are the means of four replicates ± standard error. “Whole season” includes from March 26 to November 12 for all treatments. “Forced season” involves the second cycle of the forced treatments (from forced budburst to November 12). The “Forced season” Tukey test was carried out comparing to “Whole season” Control. Significance level under “Forced season” only refers to differences between both forced treatments using ANOVA.

### 3.5 Carry-over effects

Carry-over effects from the 2018 season, such as a lower number of bunches per shoot before forcing, were detected in CF_early_ but not in CF_late_ (**Table 2**). In addition, LA before forcing was lower in CF_early_ than in the Control vines (**Figure 2A**) suggesting lower vigour in CF_early_ vines.

### 3.6 Plant water status, leaf stomatal conductance, leaf net photosynthesis and quantum yield

**Figure 3:**
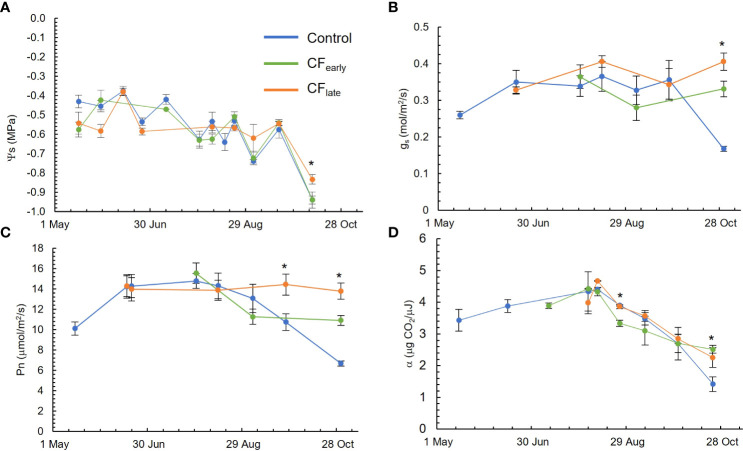
Seasonal evolution of stem water potential (Ψ_s_) **(A)**, leaf stomatal conductance at midday (g_s_) **(B)**, sunlit leaf net photosynthesis at midday (Pn) **(C)** and quantum yield (α) **(D)** in the 2019 season. Blue, green, and orange lines correspond to the Control, CF_early_ and CF_late_ treatments, respectively. Bars indicate standard deviation of eight measurements excepting for quantum yield in which three measurements were carried out. * indicates statistical differences using ANOVA (P < 0.05).

The Ψ_s_ (**Figure 3A**) was always above -0.8 MPa. The only exception was on October 10, after an irrigation failure. Measured g_s_ (**Figure 3B**) was above or close to 0.3 mol/m^2^/s throughout the growing season. The CF_late_ treatment tended to keep g_s_ nearly constant throughout the season; the same pattern was observed with Ψ_s_.

While the Control and CF_early_ treatments reached their maximum Pn rate (16 μmol/m^2^/s) in late July and then Pn began to decline, Pn was constant throughout the season for the CF_late_, at about 14 μmol/m^2^/s (**Figure 3C**). However, quantum yield followed similar patterns in the three treatments, although lower values were observed for CF_early_ on August 25 and higher values were observed for the forced vines than for the Control at the end of the season, probably because the Control leaves began to senesce (**Figure 3D**).

### 3.7 Validation of the canopy net carbon exchange model

As mentioned earlier, whole-canopy gas exchange chambers were used to validate the model. The temperature inside the chamber exceeded the ambient temperature by 7°C in exceptional cases, but rarely exceeded 35°C. The maximum vapour pressure deficit recorded in the chamber was 4.6 kPa but remained below 3.5 kPa most of the time (see **Supplementary Table 4** for more information on chamber conditions). Canopy NCE_ch_ values were between 4 and 12 μmol/m^2^/s (see more information about chamber results in **Supplementary Table 5**).

**Figure 4:**
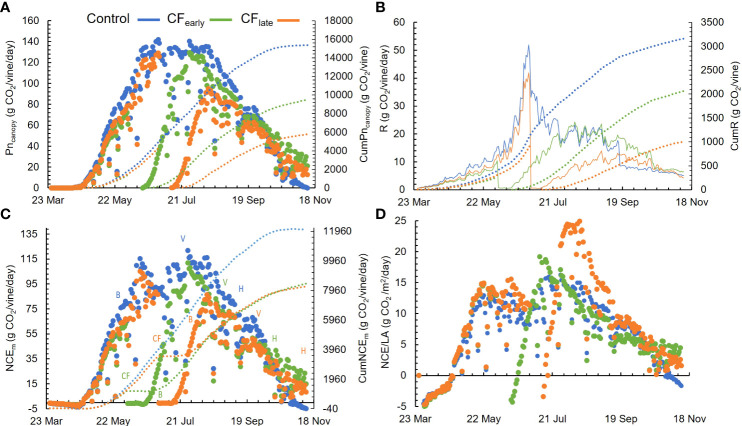
Seasonal patterns for daily modelled whole-canopy net photosynthesis (Pn_canopy_) and cumulative Pn_canopy_ (CumPn_canopy_) **(A)**, daily (R) and cumulative (CumR) above ground modelled respiration **(B)**, daily (NCE_m_) and cumulative (CumNCE_m_) net carbon exchange **(C)** and daily modelled NCE_m_ per unit of leaf area (NCE/LA) **(D)**. B, bloom; V, veraison; H, harvest. Dotted lines represent the cumulated parameters. Blue, green, and orange colours correspond to the Control, CF_early_ and CF_late_ treatments. Each plot is the average of modelling four vines for each treatment in 2019.

From May 29 to September 26, 14 different whole-canopy NCE_ch_ measurements were obtained and used to validate the model. Not all measurements included the entire day. The inputs used to run the model the same days on which the chambers were operated are summarized in **Supplementary Table 6**. The NCE_m_ correlated well with NCE_ch_ measurements (**Supplementary Figure 3**) regardless of the treatment. NCE_ch_ was well explained by the model (R^2^ = 0.95), and adequately adjusted to the 1:1 line. The NSE was 0.95 indicating a good model performance, and the error was low (RMSE = 5.8 g CO_2_/vine/day). Typical and representative days of the regional summer weather and high levels of variability in canopy size and crop phenology were covered. On average, the estimated cumulative Pn_canopy_ was 15348, 10924 and 10201 and cumulative respiration was 3159, 2335 and 1926 g CO_2_/vine for Control, CF_early,_ and CF_late_ respectively (**Figures 4A, B**).

### 3.8 Daily and seasonal carbon balance

Before forcing, Pn_canopy_ and respiration (R) did not differ between treatments (**Figures 4A, B**). However, after forcing, carbon loss by respiration was 21.8% and 16.8% for CF_early_ and CF_late_ (*P<* 0.05), respectively. NCE_m_ was 6 g CO_2_/day higher in Control than in CF_early_ when comparing the time before forcing between the Control and CF treatments. However, there were no significant differences in CF_late_, compared with Control. Note that the period before forcing in CF_early_ ranged from March 26 to June 3, whereas in CF_late_ it ranged from March 26 to July 1, 28 days longer. Forcing reduced NCE_m_ to zero or below zero for 17 (CF_early_) and 13 (CF_late_) days. CF_early_ vines reached the maximum NCE_m_ 55 days post-forcing and CF_late_ 41 days. In Control vines, NCE_m_ averaged 60.9 g CO_2_/day from budburst (March 26) to harvest (September 16). Similar NCE_m_ was observed in CF_early_ vines from forced budburst (June 14) to harvest (October 16), with an average of 55.9 g CO_2_/day (**Figure 4C**). However, from budburst to harvest (from July 11 to November 11), average NCE_m_ was lower in CF_late_ (38.7 g CO_2_/vine/day) than the other two treatments. When NCE_m_ were normalized by leaf area, higher NCE/LA was observed only for 10 days in CF_early_ vines. In contrast, CF_late_ had the highest NCE/LA values for 46 days after forcing treatments (**Figure 4D**). In the CF_late_ treatment, maximum NCE/LA was 24.5 g CO_2_/m^2^/day compared with 19.7 g CO_2_/m^2^/day in CF_early_ and 15.5 g CO_2_/m^2^/day in Control.

Pn_canopy_ and NCE_m_ began to decline after August 11 in CF_early_ (**Figure 4A**), which was due to a sharp decline in leaf photosynthesis and quantum yield (**Figures 3C, D**). This decline was milder in Control vines and delayed until September 8 in CF_late_. During berry ripening, average NCE_m_ levels differed among the three treatments (*P<* 0.05). Higher NCE_m_ levels were observed in Control (85.1 g CO_2_/day) than in CF_early_ (45.8 g CO_2_/day) and CF_late_ (19.7 g CO_2_/day) (**Figure 4C**).

**Figure 5:**
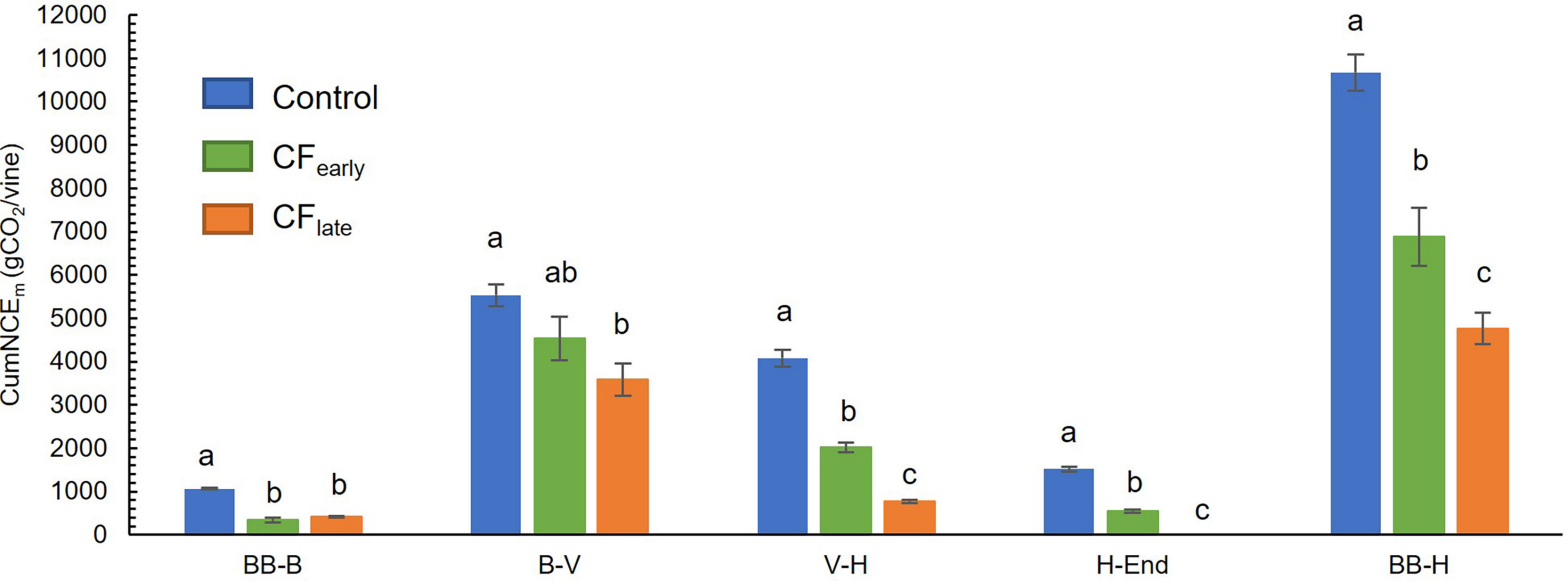
Cumulative net carbon exchange (CumNCE_m_) between two different phenological stages in 2019. Forced treatments included only post-forcing data. BB, bud burst; B, bloom; FP, forcing pruning; V, Veraison; H, harvest; End, end of season. Blue, green, and orange correspond to Control, CF_early_ and CF_late_ treatments, respectively. Each column indicates the mean of four vines. Bars indicate standard deviation. Treatment effects were analysed using ANOVA and the means were separated with the Tukey test. Means followed by different letters are significantly different at *P* < 0.05.

Throughout the season, CumNCE_m_ was higher in Control vines than in the two forced treatments, which accumulated a similar amount of carbon between them (**Figure 4C**). Prior to forcing, CF_late_ vines accumulated 37% more carbon than CF_early_ vines. However, after forcing, lower CumNCE_m_ was found in CF_late_ vines than CF_early_ (33%). From budburst (after forcing in the forced treatments) to harvest, CumNCE_m_ differed greatly between treatments, with values of 10672 g CO_2_/vine in Control, 6883 g CO_2_/vine in CF_early_ and 4763 g CO_2_/vine in CF_late_ (**Figure 5**). Forced vines reduced CumNCE by 60-70% from budburst to full bloom. From veraison to harvest, CF_late_ vines accumulated 60% less carbon than CF_early_ and 81% less than Control vines (**Figure 5**).

### 3.9 Carbohydrate reserves

At budburst, TNSC_initial_ was lower in CF_early_ vines (**Table 5**). At forcing dates, both forced treatments reduced TNSC (0.9 %DW CF_early_ and 8.2 %DW CF_late_) and RNSC (2.2 %DW CF_early_ and 1 %DW CF_late_) compared to pre-forcing budburst level (March 26). Moreover, at forcing dates, forced vines had lower TNSC than the Control vines at budburst, and TNSC was lower in CF_late_ than in CF_early_. At harvest, no differences in RNSC were observed between treatments, which may be attributable to the high variability of the Control treatment. However, in CF_early_ RNSC was 20% higher than in Control and 18% higher than in CF_late_. From forcing to harvest, CF_early_ increased both TNSC (6.2 %DW) and RNSC (5 %DW) whereas CF_late_ increased TNSC (7.7 %DW) but slightly decreased RNSC (0.8 %DW).

**Table 5:** Total non-structural carbohydrate concentration in trunk (TNSC) and roots (RNSC) at budburst (initial), forcing date (forcing) and at harvest (harvest) for Control and forced treatments in 2019.

Parameter	Control	CF_early_	CF_late_	Significance level
TNSC_initial_ (%DW)	16.0 ± 0.3 a	12.3 ± 0.1 b	16.3 ± 1.1 a	*
TNSC_forcing_ (%DW)	16.0 ± 0.3 a	11.2 ± 0.9 b	8.1 ± 0.6 c	*
TNSC_harvest_ (%DW)	20.2 ± 0.2	17.4 ± 3.1	15.8 ± 2.3	ns
RNSC_initial_ (%DW)	37.2 ± 4.6	34.6 ± 1.3	32.5 ± 3.3	ns
RNSC_forcing_ (%DW)	37.2 ± 4.6	32.4 ± 1.1	31.5 ± 2.3	ns
RNSC_harvest_ (%DW)	29.7 ± 6.5	42.7 ± 0.3	30.7 ± 3.6	ns(*)

Treatment effects were analysed using ANOVA and the means were separated with the Tukey test. Means followed by different letters are different at P< 0.05. Significance levels in brackets correspond to differences only analysing forced treatments. ns, not significant. *=significant. TNSC_initial_ and RNSC_initial_ was sampled on April 2 for all treatments. For the Control treatment, TNSC_forcing_= TNSC_initial_ and RNSC_forcing_ = RNSC_initial_. TNSC data are means of three vines ± standard.

## 4 Discussion

### 4.1 Leaf area and light interception

In contrast to the CF_late_ vines, after forcing, the CF_early_ vines recovered leaf area and daily fraction of intercepted radiation that were comparable to those of the Control (**Figures 2A, B**). However, leaf and shoot removal, lateral trimming and hedging were performed on the Control vines but not on the forced treatments, limiting potential vegetative growth on the Control vines. These observations are consistent with other studies reporting reduced vegetative growth in forced vines (Gu et al., 2012; Martinez De Toda et al., 2019; Martínez-Moreno et al., 2019), and the later the forcing occurred, the lower the leaf area (Martinez De Toda et al., 2019). Reduced growth performance in forced treatments has been attributed to lower carbon reserve status in forced vines (Martínez-Moreno et al., 2019) as early growth stages are dependent on carbon reserves (Smith and Holzapfel, 2009; Holzapfel et al., 2010). Our observation of lower TNSC at forcing date (**Table 5**) and lower vigour (**Figure 2A**) in CF_late_ than in CF_early_, supports this hypothesis.

### 4.2 Vine water status and leaf physiological parameters

We did not observe a significant difference in water status between treatments. Stem water potential was above the threshold of -0.8 MPa that we established to avoid limiting photosynthesis (Marsal et al., 2008). In addition, leaf stomatal conductance was greater than 0.3 mol/m^2^/s, which did not limit leaf net photosynthesis (Escalona et al., 1999). Therefore, we excluded water stress as a cause of photosynthesis limitation. Because younger leaves (less than 100 days) have higher photosynthetic rates than older leaves (Poni et al., 1994), we expected higher leaf net photosynthesis rates in both forced treatments. In CF_late_, leaf net photosynthetic rates were constant until October (**Figure 3C**), indicating that leaves remained more active. In contrast, this effect was not observed in CF_early_ leaves (**Figure 3C**). In vines with a high LA/fruit ratio, leaf net photosynthesis tends to decrease (Iacono et al., 1995), which may be attributed to feedback inhibition in response to low sink activity (Paul and Foyer, 2001). Therefore, feedback inhibition may explain the lower leaf net photosynthesis observed in CF_early_ leaves because the LA/fruit ratio was extremely high. The quantum yield levels we measured (**Figure 3D**) were in the range (0 to 4 μg CO_2_/J) of those previously reported for grapevine (Poni et al., 2006; Mirás-Avalos et al., 2018). However, more significant differences in the seasonal quantum yield patterns due to the treatments were expected.

### 4.3 Net carbon exchange NCE model (NCE_m_) and carbohydrate reserves

#### 4.3.1 Validation of the model

The model showed high correlation with the chamber measurements and had an acceptable error (**Supplementary Figure 3**). Modelled and measured NCE values were within the range of values reported by other studies on Cabernet Sauvignon (Poni et al., 2006) and Tempranillo (Pagay, 2016). Total above ground respiration losses were 23.2%, 20.7% and 18.4% of the Pn_canopy_ for the Control, CF_early,_ and CF_late_, respectively (**Figures 4A, B**). These values are close to the 25% reported for Cabernet Sauvignon (Poni et al., 2006) and the 19.3% for Tempranillo cultivar (Medrano et al., 2015). Therefore, the model can be considered a realistic tool for carbon balance analysis.

#### 4.3.2 Whole canopy net carbon exchange dynamics

As we suspected, the crop forcing technique affected daily and seasonal net carbon exchange patterns. The capacity to accumulate carbon in a forced season (from forced budburst to November 12) is reduced (**Figure 4C**) mainly because forced seasons are shorter than non-forced. In addition, photosynthesis ceased in forced vines due to the forcing, and a recovery period of approximately 45 days was required to reach maximum canopy photosynthesis and net carbon exchange (NCE_m_) rates (**Figure 4C**). Since whole-canopy net carbon exchange is proportional to the radiation intercepted by the canopy (Poni et al., 2003; Petrie et al., 2009) maximum daily NCE_m_ was recovered in CF_early_ but not in CF_late_ (**Figure 4D**), which also negatively affected the cumulative NCE_m_ for the forced season (**Figure 5**).

The initial FIPAR and LA in 2019 were slightly greater in Control compared to the forced treatments (**Figures 2A, B**). This might have biased the result presented in **Figure 5**. A larger LA at the begging of the season should result in a larger NCE_m_ before the forcing. However, we found non-significant differences in LA and FIPAR (**Figures 2A, B**) from June to CF_late_ forcing date. Besides, the trunk diameter of all vines was measured right before the begging of the experiment in 2017. Trunk diameters were 38.0 cm (Control), 41.2 cm (CF_early_) and 40.9 cm (CF_late_). No statistical differences were observed between treatments *(P =* 0.28) (**Supplementary Table 1**). The treatments were severe enough (removal of all leaves and bunches) to overcome the experimental error in LA and FIPAR. All in all, confirms that plants presented the same vigour at the beginning of the experiment. Therefore, the differences observed in **Figure 5** were attributed to the treatments imposed than to an experimental error.

In a fruit removal experiment, net carbon exchange at a canopy level was reduced in vines without fruits compared to vines with fruit load (Petrie et al., 2000). Because leaf net photosynthesis is an input to the whole canopy net carbon exchange model, the decline in leaf net photosynthesis observed in CF_early_, which we attributed to the extremely high LA/fruit ratio, was also observed by the whole-canopy model (**Figure 4C**). In a forced double cropping experiment, NCE/LA was reported to be higher in forced vines than in unforced vines due to the younger canopy (Poni et al., 2021). However, these vines had a LA/fruit ratio of 2 m^2^/kg because primary crop was not removed at forcing, which is close to the LA/fruit ratio we observed in CF_late_ but significantly lower than that observed in CF_early_ (**Table 2**). Therefore, after vegetative growth slowed down in CF_early_ (in August), the sink activity of the fruit was not sufficient to maintain a high NCE_m_ rate and, NCE/LA was similar to the Control despite the younger canopy (**Figures 4C, D**). On the other hand, respiration (R) is a function of biomass (Amthor, 2000). By partitioning a lower proportion of biomass to shoots and maintaining the proportion of leaves (**Table 4**), CF_late_ vines improved the daily carbon balance and increased the photosynthesis/respiration ratio. Thus, higher NCE/LA for CF_late_ (**Figure 4D**), at least until mid-October when CF_early_ vines lost some of LA (**Figure 2A**) increasing NCE/LA for this treatment, could therefore be explained by a higher proportion of sunlit leaves due to a sparser canopy, improved daily carbon balance and to a higher leaf photosynthetic activity. In a late-pruning experiment, vines in which budburst was delayed by 31 days, the same delay we observed between CF_early_ and CF_late_, were able to compensate for the accumulated carbon budget of traditionally managed vines by increasing the NCE/LA ratio (Gatti et al., 2016). In our experiment, higher canopy efficiency in CF_late_ (**Figure 4D**) could not compensate for the carbon accumulation (**Figure 5**). This was partly because canopy photosynthesis was limited by the reduced leaf area of the vines but also because solar radiation and daylength decreased rapidly from September onward (**Figure 1A**). Delaying harvest until mid-November therefore drastically reduces the capacity to gain carbon from veraison to harvest (**Figure 5**), although CF_late_ vines could reach TSS comparable to the other treatments (**Table 1**) likely using previously accumulated carbohydrate reserves.

#### 4.3.3 Carbohydrate reserves: trunk and root non-structural carbohydrates

Regarding the carbohydrate reserve analysis, the concentrations observed in trunk and roots were close to other values reported for fully irrigated vines in hot climates (maximum TNSC of 20 %DW and RNSC of 40 %DW) (Smith and Holzapfel, 2009; Holzapfel et al., 2010). The lower carbohydrate reserves observed at forcing dates compared to the time of budburst (March 26) (**Table 5**) indicated that the NCE accumulated prior to forcing was insufficient to replenish carbohydrates reserves and, therefore, the capacity to refill carbohydrate storage at the whole-vine level depends mainly on the photosynthetic capacity from forcing to the end of season. These results are consistent with previous reports in which the minimum of carbohydrate reserves was observed between full bloom and one month later (Zapata et al., 2004; Bennett et al., 2005; Holzapfel et al., 2010; Zufferey et al., 2012) and are supported by the fact that, although cumulative net carbon exchange was the same for both forced treatments for the whole season (**Figure 4C**), the RNSC was lower at harvest for CF_late_ because the capacity to accumulate carbon is reduced in the CF_late_ forced season (**Figure 5**). However, LA/fruit indicates a highly favourable source:sink relationship for forced vines, especially for CF_early_ (**Table 2**), which can be confirmed by calculating NCE_m_/yield (3.6 g CO_2_/g yield for Control, 9.14 g CO_2_/g yield for CF_early_, and 7.84 g CO_2_/g yield CF_late_). As a result, at harvest, forced treatments increased carbohydrate reserves (CF_early_) or maintained them (CF_late_) compared to pre-forcing budburst (sampled on April 2) (**Table 5**), compensating for the lower seasonal cumulative NCE_m_ (**Figure 4C**). The large increase in carbohydrate reserves from forced budburst to harvest in the CF_early_ treatment supports the hypothesis of feedback inhibition of photosynthesis acting carbohydrate storage organs as major carbon sinks, despite being among the lowest priority sink (Minchin and Lacointe, 2005). On the other hand, the Control treatment reduced RNSC by 8% compared to budburst (**Table 5**), which is in accordance with other studies which revealed that carbon reserves in roots, are very sensitive to source:sink relationships and fruit yield (Smith and Holzapfel, 2009; Zufferey et al., 2012).

### 4.4 Agronomic implications of the crop forcing technique related to carbon availability

In 2018, the CF_late_ treatment was applied too late and did not reach the target TSS (**Table 1**). However, in our experiment, the main objective of the crop forcing technique which was delaying berry ripening in a cooler environment (**Figure 1A**) and harvesting at the desired TSS but higher acidity, was achieved, although yield was reduced (**Table 1**). Under different environmental conditions, with different cultivars and irrigation strategies, a reduced bunch weight, due to a lower number of berries per bunch and smaller berry size, is a characteristic of forced vines (Gu et al., 2012; Lavado et al., 2019; Martinez De Toda et al., 2019; Martínez-Moreno et al., 2019; Pou et al., 2019) which is consistent with our results (**Table 1**). Low carbon availability at the beginning of the berry development is crucial for the final berry weight, since an imbalance at that time cannot be compensated later (Candolfi-Vasconcelos and Koblet, 1990). Besides, the number of berries per bunch is highly sensitive to carbon stress (Bennett et al., 2005), as carbohydrates are required for proper flower formation (Lebon et al., 2008) although elevated temperatures around budburst may also have the same effect (Petrie and Clingeleffer, 2005; Pagay and Collins, 2017). In addition to the observed decrease in trunk carbon reserves at forcing (**Table 5**), the period from budburst to full bloom was reduced from 65 days in the Control to 23 days in the forced treatments (**Figure 1B**), resulting in a reduced capacity to provide carbon (**Figure 5**) for flower formation and the first stages of the berry development. However, our results do not allow to distinguish between carbon or temperature stress or, the most likely hypothesis, a combination of both stress factors as the cause of the large decrease in bunch weight. Since bunch compactness was not reduced in the forced treatments (**Table 2**), we ruled out a reduction in fruit set percentage.

In the crop forcing technique, vines that are forced early in the season (e.g. around bloom and fruit set) are generally more sensitive to a reduction in the number of bunches per shoot suggesting an incomplete formation of the inflorescence primordia (Gu et al., 2012; Martinez De Toda et al., 2019; Martínez-Moreno et al., 2019). This is consistent with our observations of a reduced number of bunches per shoot in CF_early_ but not in CF_late_ in 2019 (**Tables 1** and **2**). However, we did not observe the same effect in the season 2018 probably because the treatment was applied 11 days later than in 2019. Therefore, the timing of forcing is a relevant aspect in forcing performance as reported in previous experiments (Martinez De Toda et al., 2019; Martínez-Moreno et al., 2019). In addition, in forcing treatments, inflorescence formation, which is highly sensitive to carbon availability (Keller and Koblet, 1995; Sánchez and Dokoozlian, 2005; Lebon et al., 2008), occurred partly before forcing and partly after forcing, shortly after carbon assimilation ceased. Vines forced earlier have a lower capacity to accumulate carbon before forcing (**Figure 4C**), and after a high yield season, as was the case with CF_early_ in 2018 (**Table 1**), the level of carbohydrate reserves may be lower at non-forced budburst (**Table 5**). Note that CF_early_ reduced the number of bunches per shoot even before the forcing treatment (**Table 2**). Therefore, the state of carbon reserves at the time of non-forced budburst, which depends on the photosynthetic capacity and source-sink relationship in the previous year (Lebon et al., 2008) and at forcing, is a determining factor for the number of forced bunches per shoot.

Since biomass in forced vines was quite conservative from year to year (**Table 3**), an increase in yield implies a decrease in pruning weight (**Table 1**), which is closely related to vine photosynthetic capacity (Kliewer and Dokoozlian, 2005). Therefore, high yields in forced vines must lead to a reduction in carbon reserves for the following season because both, reduced carbon assimilation and a stronger fruit sink activity. However, in a climate change scenario, increased temperatures extend the growing season. Together with the expected increase in ambient CO_2_ concentration, higher photosynthetic capacity of vines as well as higher yields and vine vigour are expected (Kizildeniz et al., 2015). The yield reduction associated to the crop forcing technique could be milder and wine producers could consider crop forcing as a less risky tool to produce high quality wines. On the other hand, the crop forcing technique is already a valuable tool for research. Recently, this technique has been successfully used to study and validate the robustness of vine development models (Prats-Llinàs et al., 2020).

As far as we know, there is only one other technique that can delay harvest by two months on grapevines. This technique, double cropping, is a version of forced regrowth in which the primary crop and the leaves of the six basal nodes are retained at the time of forcing. It was validated in Pinot Noir in two consecutive years, (Poni et al., 2021) and in Tempranillo in only one season (Martínez De Toda, 2021) demonstrating that dormant buds are capable to break dormancy without removing all the leaves. Double cropping overcame the yield reduction observed with the crop forcing technique (Lavado et al., 2019; Martinez De Toda et al., 2019; Martínez-Moreno et al., 2019; Pou et al., 2019), as the sum of primary and forced yields was even higher than in the non-forced vines (Martínez De Toda, 2021; Poni et al., 2021). Moreover, the carbon balance of the vine was even improved in forced vines as the leaf area was substantially restored with younger leaves, which ended up in a more functional canopy than the canopy of the unforced vines (Poni et al., 2021). In addition, unlike our experiment in which NCE_m_ was reduced to zero for about two weeks after forcing (**Figure 4C**), in Poni et al. (2021) the reduction of NCE soon after forcing was only about 55% of the pre-forcing NCE. Therefore, the retained basal leaves continued to provide sugars to the entire vine during the interval between forcing and the emergence of new functional leaf area which, probably, helped to drive regrowth of the forced shoots and minimize the depletion of carbohydrate reserves of the permanent organs of the vine. It is relevant that, unlike our experiment for CF_early_ treatment, no carry-over effects were observed in vines in which double cropping was applied, which can be related to a better vine carbohydrate status soon after forcing. However, it should be noted that the experiment of Poni et al. (2021) was conducted with an early cultivar following the quality criteria for sparkling wine, on potted vines and with site-specific conditions different from those of our experiment. Therefore, further research is necessary to explore the promising benefits of retaining leaves and fruits on forced vines in medium to late ripening cultivars and in field conditions.

## 5 Conclusions

Our study confirms that the forcing technique has a negative impact on the seasonal carbon balance under our experimental conditions. The shorter season, smaller vines, and the environmental conditions at the end of the season limit the seasonal carbon balance. However, the capacity to restore carbohydrate reserves after forcing was demonstrated in early and late forcing dates. Therefore, the yield reduction seems to be a necessary strategy to increase the source:sink ratio, allowing the forced vines to restore carbon reserves at the whole-canopy level. Because the state of carbon reserves before and at the time of forcing plays an important role in forced yield, techniques that can modulate carbon reserve dynamics applied before forcing, such as mild water stress or sink organs removal (e.g. fruit removal) would be of interest for improving the carbon availability in the forced season. However, after forcing, any viticultural practises that restrict carbon assimilation and vegetative growth must be used with caution, as they may affect the carbon reserves for the next season. Of particular interest is the possibility of not removing all the leaves when vines are forced to provide carbon for shoot regrowth, as well as not removing all the bunches to counteract the drastic reduction in yield of the forced vines to make this technique more acceptable to winegrowers.

## Data availability statement

The raw data supporting the conclusions of this article will be made available by the authors, without undue reservation.

## Author contributions

JG and OG-T conceived, planned, and supervised this study. JO-M contributed to the planning of the experiment. JO-M and MA developed the net carbon exchange chambers and realized most of the field measurements and tasks. JO-M did the processing and analysis of all the data and drafted and finalized the manuscript. JG, OG-T and MA reviewed the manuscript. All authors contributed to the article and approved the submitted version.

## Funding

This work was supported by funds from the Instituto Nacional de Investigación y Tecnología Agraria y Alimentaria (INIA) research project RTA2015-00089-C02-02 and from the H2020 programme project Vineyards´ Integrated Smart Climate. Application (VISCA) 730253. The participation of Jori Oliver-Manera was founded by the Ministerio de Ciencia e Innovación in an Instituto Nacional de Investigación fellowship BES-2017-082089.

## Acknowledgments

The authors would like to thank Mercè Mata, Jesús del Campo, Pinelopi Mavropouli, Konstantina Moschou, Arnau Roig, Ana Pelechá and Christian Jorfré for their hard work in the field and Dr Jaume Casadesús and Dr Joaquim Bellvert for their scientific advice. Special thanks to Raïmat Wineries for their support throughout the experiment.

## Conflict of interest

The authors declare that the research was conducted in the absence of any commercial or financial relationships that could be construed as a potential conflict of interest.

## Publisher’s note

All claims expressed in this article are solely those of the authors and do not necessarily represent those of their affiliated organizations, or those of the publisher, the editors and the reviewers. Any product that may be evaluated in this article, or claim that may be made by its manufacturer, is not guaranteed or endorsed by the publisher.

## References

[B1] Allen M. R. Dube O. P. Solecki W. Aragón-Durand F. Cramer W. Humphreys S.. (2018). “Framing and Context,” In: Global Warming of 1.5°C. An IPCC Special Report on the impacts of global warming of 1.5°C above pre-industrial levels and related global greenhouse gas emission pathways, in the context of strengthening the global response to the threat of climate change, sustainable development, and efforts to eradicate poverty. Eds. Masson-Delmotte V. Zhai P. Pörtner H.-O. Roberts D. Skea J. Shukla P. R. (Cambridge, UK and New York, NY, USA: Cambridge University Press). pp. 49–92. doi: 10.1017/9781009157940.003

[B2] Allen R. G. Pereira L. S. Raes D. Smith M. (1998). Crop Evapotranspiration: Guidelines for Computing Crop Water Requirements: Irrigation and Drainage Paper No. 56. Rome: FAO.

[B3] Amthor J. S. (2000). The McCree-de wit-penning de Vries-thornley respiration paradigms: 30 years later. Ann. Bot. 86, 1–20. doi: 10.1006/anbo.2000.1175

[B4] Bellvert J. Mata M. Vallverdú X. Paris C. Marsal J. (2020). Optimizing precision irrigation of a vineyard to improve water use efficiency and profitability by using a decision-oriented vine water consumption model. Precis. Agric 22, 319–41. doi: 10.1007/s11119-020-09718-2

[B5] Bennett J. Jarvis P. Creasy G. L. Trought M. C. T. (2005). Influence of defoliation on overwintering carbohydrate reserves, return bloom, and yield of mature chardonnay grapevines. Am. J. Enol. Vitic. 56, 386–393. doi: 10.5344/ajev.2005.56.4.386

[B6] Buesa I. Caccavello G. Basile B. Merli M. C. Poni S. Chirivella C.. (2019). Delaying berry ripening of bobal and tempranillo grapevines by late leaf removal in a semi-arid and temperate-warm climate under different water regimes. Aust. J. Grape Wine Res. 25, 70–82. doi: 10.1111/ajgw.12368

[B7] Buysse J. Merckx R. (1993). An improved colorimetric method to quantify sugar content of plant tissue. J. Exp. Bot. 44, 1627–1629. doi: 10.1093/jxb/44.10.1627

[B8] Candolfi-Vasconcelos M. C. Koblet W. (1990). Yield, fruit quality, bud fertility and starch reserves of the wood as a function of leaf removal in *Vitis vinifera* - evidence of compensation and stress recovering. Vitis 29, 199–221.

[B9] Charles-Edwards D. A. (1982). Physiological determinants of crop growth (London: Academic Press).

[B10] Coombe B. G. (1995). Growth stages of the grapevine: Adoption of a system for identifying grapevine growth stages. Aust. J. Grape Wine Res. 1, 104–110. doi: 10.1111/j.1755-0238.1995.tb00086.x

[B11] Corelli-Grappadelli L. Magnanini E. (1993). A whole-tree system for gas-exchange studies. HortScience 28, 41–45. doi: 10.21273/HORTSCI.28.1.41

[B12] Escalona J. M. Flexas J. Medrano H. (1999). Stomatal and non-stomatal limitations of photosynthesis under water stress in field-grown grapevines. Aust. J. Plant Physiol. 26, 421–433. doi: 10.1071/PP99019

[B13] Escalona J. M. Tomàs M. Martorell S. Medrano H. Ribas-Carbo M. Flexas J. (2012). Carbon balance in grapevines under different soil water supply: Importance of whole plant respiration. Aust. J. Grape Wine Res. 18, 308–318. doi: 10.1111/j.1755-0238.2012.00193.x

[B14] Gatti M. Pirez F. J. Chiari G. Tombesi S. Palliotti A. Merli M. C.. (2016). Phenology, canopy aging and seasonal carbon balance as related to delayed winter pruning of *Vitis vinifera* L. cv. sangiovese grapevines. Front. Plant Sci. 7, 659. doi: 10.3389/fpls.2016.00659 27242860 PMC4865496

[B15] Gu S. Jacobs S. D. McCarthy B. S. Gohil H. L. (2012). Forcing vine regrowth and shifting fruit ripening in a warm region to enhance fruit quality in “Cabernet sauvignon“ grapevine (*Vitis vinifera* L.). J. Hortic. Sci. Biotechnol. 87, 287–292. doi: 10.1080/14620316.2012.11512866

[B16] Gutiérrez-Gamboa G. Zheng W. de Toda F. M. (2021). Current viticultural techniques to mitigate the effects of global warming on grape and wine quality: A comprehensive review. Food Res. Int. 139. doi: 10.1016/j.foodres.2020.109946 33509499

[B17] Hernández-Montes E. Escalona J. M. Tomàs M. Medrano H. (2020). Plant water status and genotype affect fruit respiration in grapevines. Physiol. Plant 169, 544–554. doi: 10.1111/ppl.13093 32187689

[B18] Holzapfel B. P. Smith J. P. Field S. K. James Hardie W. (2010). Dynamics of carbohydrate reserves in cultivated grapevines. Hortic. Rev. (Am. Soc Hortic. Sci). 37, 143–211. doi: 10.1002/9780470543672.ch3

[B19] Iacono F. Bertamini M. Scienza A. Coombe B. G. (1995). Differential effects of canopy manipulation and shading of *Vitis vinifera* L. cv. Cabernet sauvignon. leaf gas exchange, photosynthetic electron transport rate and sugar accumulation in berries. Vitis - J. Grapevine Res. 34, 201. doi: 10.1080/01904169509365023

[B20] Keller M. (2010). Managing grapevines to optimise fruit development in a challenging environment: A climate change primer for viticulturists. Aust. J. Grape Wine Res. 16, 56–69. doi: 10.1111/j.1755-0238.2009.00077.x

[B21] Keller M. Koblet W. (1995). Dry matter and leaf area partitioning, bud fertility and second season growth of *Vitis vinifera* L.: Responses to nitrogen supply and limiting irradiance. Vitis 34, 77–83.

[B22] Kizildeniz T. Mekni I. Santesteban H. Pascual I. Morales F. Irigoyen J. J. (2015). Effects of climate change including elevated CO2 concentration, temperature and water deficit on growth, water status, and yield quality of grapevine (*Vitis vinifera* L.) cultivars. Agric. Water Manage. 159, 155–164. doi: 10.1016/j.agwat.2015.06.015

[B23] Kliewer W. M. Dokoozlian N. K. (2005). Leaf area/crop weight ratios of grapevines: Influence on fruit composition and wine quality. Am. J. Enol. Vitic. 56, 170–181. doi: 10.5344/ajev.2005.56.2.170

[B24] Landhäusser S. M. Chow P. S. Turin Dickman L. Furze M. E. Kuhlman I. Schmid S.. (2018). Standardized protocols and procedures can precisely and accurately quantify non-structural carbohydrates. Tree Physiol. 38, 1764–1778. doi: 10.1093/treephys/tpy118 30376128 PMC6301340

[B25] Lavado N. Uriarte D. Mancha L. A. Moreno D. Valdés E. Prieto M. H. (2019). Effect of forcing vine regrowth on “Tempranillo“ (*Vitis vinifera* L.) berry development and quality in extremadura. Vitis - J. Grapevine Res. 58, 135–142.

[B26] Lebon G. Wojnarowiez G. Holzapfel B. Fontaine F. Vaillant-Gaveau N. Clément C. (2008). Sugars and flowering in the grapevine (*Vitis vinifera* L.). J. Exp. Bot. 59, 2565–2578. doi: 10.1093/jxb/ern135 18508810

[B27] Lopes C. Pinto P. A. (2005). Easy and accurate estimation of grapevine leaf area with simple mathematical models. Vitis - J. Grapevine Res. 44, 55–61. doi: 10.5073/vitis.2005.44.55-61

[B28] Marsal J. Mata M. Del Campo J. Arbones A. Vallverdú X. Girona J.. (2008). Evaluation of partial root-zone drying for potential field use as a deficit irrigation technique in commercial vineyards according to two different pipeline layouts. Irrig. Sci. 26, 347–356. doi: 10.1007/s00271-007-0098-4

[B29] Martínez De Toda F. (2021). Global warming allows two grape crops a year, with about two months apart in ripening dates and with very different grape composition-the forcing vine regrowth to obtain two crops a year. Vitis - J. Grapevine Res. 60, 119–124. doi: 10.5073/vitis.2021.60.119-124

[B30] Martinez De Toda F. Garcia J. Balda P. (2019). Preliminary results on forcing vine regrowth to delay ripening to a cooler period. Vitis - J. Grapevine Res. 58, 17–22. doi: 10.5073/vitis.2019.58.17-22

[B31] Martínez-Moreno A. Sanz F. Yeves A. Gil-Muñoz R. Martínez V. Intrigliolo D. S.. (2019). Forcing bud growth by double-pruning as a technique to improve grape composition of *Vitis vinifera* L. cv. tempranillo in a semi-arid Mediterranean climate. Sci. Hortic. (Amsterdam). 256, 108614. doi: 10.1016/j.scienta.2019.108614

[B32] Medrano H. Tomás M. Martorell S. Flexas J. Hernández E. Rosselló J.. (2015). From leaf to whole-plant water use efficiency (WUE) in complex canopies: Limitations of leaf WUE as a selection target. Crop J. 3, 220–228. doi: 10.1016/j.cj.2015.04.002

[B33] Minchin P. E. H. Lacointe A. (2005). New understanding on phloem physiology and possible consequences for modelling long-distance carbon transport. New Phytol. 166, 771–779. doi: 10.1111/j.1469-8137.2005.01323.x 15869640

[B34] Mira de Orduña R. (2010). Climate change associated effects on grape and wine quality and production. Food Res. Int. 43, 1844–1855. doi: 10.1016/j.foodres.2010.05.001

[B35] Mirás-Avalos J. M. Uriarte D. Lakso A. N. Intrigliolo D. S. (2018). Modeling grapevine performance with ‘VitiSim’ a weather-based carbon balance model: Water status and climate change scenarios. Sci. Hortic. (Amsterdam). 240, 561–571. doi: 10.1016/j.scienta.2018.06.065

[B36] Oyarzun R. A. Stöckle C. O. Whiting M. D. (2007). A simple approach to modeling radiation interception by fruit-tree orchards. Agric. For. Meteorol. 142, 12–24. doi: 10.1016/j.agrformet.2006.10.004

[B37] Pagay V. (2016). Effects of irrigation regime on canopy water use and dry matter production of ‘ tempranillo ’ grapevines in the semi-arid climate of southern Oregon , USA. Agric. Water Manage. 178, 271–280. doi: 10.1016/j.agwat.2016.10.014

[B38] Pagay V. Collins C. (2017). Effects of timing and intensity of elevated temperatures on reproductive development of field-grown Shiraz grapevines. Oeno One 51, 409–421. doi: 10.20870/oeno-one.2017.51.4.1066

[B39] Palliotti A. Cartechini A. Silvestroni O. Mattioli S. (2005). Respiration activity in different above-ground organs of *Vitis vinifera* L. in response to temperature and developmental stage. Acta Hortic. 689, 159–166. doi: 10.17660/ActaHortic.2005.689.16

[B40] Palliotti A. Tombesi S. Silvestroni O. Lanari V. Gatti M. Poni S. (2014). Changes in vineyard establishment and canopy management urged by earlier climate-related grape ripening: A review. Sci. Hortic. (Amsterdam). 178, 43–54. doi: 10.1016/j.scienta.2014.07.039

[B41] Paul M. J. Foyer C. H. (2001). Sink regulation of photosynthesis. J. Exp. Bot 52, 1383–1400. doi: 10.1093/jexbot/52.360.1383 11457898

[B42] Petrie P. R. Clingeleffer P. R. (2005). Effects of temperature and light (before and after budburst) on inflorescence morphology and flower number of Chardonnay grapevines (*Vitis vinifera* L.). Aust. J. Grape Wine Res. 11, 59–65. doi: 10.1111/j.1755-0238.2005.tb00279.x

[B43] Petrie P. R. Trought M. C. T. Howell G. S. (2000). Influence of leaf ageing, leaf area and crop load on photosynthesis, stomatal conductance and senescence of grapevine (*Vitis vinifera* L. cv. pinot noir) leaves. Vitis 39, 31–36. doi: 10.5344/ajev.2009.60.2.173

[B44] Petrie P. R. Trought M. C. T. Howell G. S. Buchan G. D. Palmer J. W. (2009). Whole-canopy gas exchange and light interception of vertically trained *Vitis vinifera* L. under direct and diffuse light. Am. J. Enol. Vitic. 60, 173–182. doi: 10.5344/ajev.2009.60.2.173

[B45] Poni S. Del Zozzo F. Santelli S. Gatti M. Magnanini E. Sabbatini P.. (2021). Double cropping in *Vitis vinifera* L. cv. pinot noir: agronomical and physiological validation. Aust. J. Grape Wine Res. 27, 508–518. doi: 10.1111/ajgw.12507

[B46] Poni S. Gatti M. Tombesi S. Squeri C. Sabbatini P. Rodas N. L.. (2020). Double cropping in *Vitis vinifera* L. pinot noir: Myth or reality? Agronomy 10, 799. doi: 10.3390/agronomy10060799

[B47] Poni S. Intrieri C. Silvestroni O. (1994). Interactions of leaf age, fruiting, and exogenous cytokinins in sangiovese grapevines under non-irrigated conditions. I.Gas exchange. Am. J. Enol. Vitic. 45, 71–78.

[B48] Poni S. Magnanini E. Bernizzoni F. (2003). Degree of correlation between total light interception and whole-canopy net CO2 exchange rate in two grapevine growth systems. Aust. J. Grape Wine Res. 9, 2–11. doi: 10.1111/j.1755-0238.2003.tb00226.x

[B49] Poni S. Palliotti A. Bernizzoni F. (2006). Calibration and evaluation of a STELLA software-based daily CO2 balance model in *Vitis vinifera* l. J. Am. Soc Hortic. Sci. 131, 273–283. doi: 10.21273/JASHS.131.2.273

[B50] Pou A. Balda P. Albacete A. Martínez De Toda F. (2019). Forcing vine regrowth to delay ripening and its association to changes in the hormonal balance. Vitis - J. Grapevine Res. 58, 95–101. doi: 10.5073/vitis.2019.58.special-issue.95-101

[B51] Prats-Llinàs M. T. Marsal J. Girona J. (2017). “Variación de la fenología, posibles efectos sobre El cultivo de la vid Chardonnay frente la climatología cambiante y sus efectos sobre la demanda hídrica,” in AERYD XXXV congreso nacional de riegos(Tarragona: AERYD) 2017, 1–6. doi: 10.25028/CNRiegos.2017.A19

[B52] Prats-Llinàs M. T. Nieto H. DeJong T. M. Girona J. Marsal J. (2020). Using forced regrowth to manipulate Chardonnay grapevine (*Vitis vinifera* L.) development to evaluate phenological stage responses to temperature. Sci. Hortic. (Amsterdam). 262, 109065. doi: 10.1016/j.scienta.2019.109065

[B53] Ramos M. C. Jones G. V. Yuste J. (2018). Phenology of tempranillo and cabernet-sauvignon varieties cultivated in the ribera del duero DO: Observed variability and predictions under climate change scenarios. Oeno One 52, 31–44. doi: 10.20870/oeno-one.2018.52.1.2119

[B54] Russell G. Jarvis P. G. Monteith J. L. (1989). Absorption of radiation by canopies and stand growth. In: Plant Canopies. pp, 21–39. doi: 10.1017/CBO9780511752308.003

[B55] Sadras V. O. Moran M. A. (2012). Elevated temperature decouples anthocyanins and sugars in berries of Shiraz and Cabernet franc. Aust. J. Grape Wine Res. 18, 115–122. doi: 10.1111/j.1755-0238.2012.00180.x

[B56] Sánchez L. A. Dokoozlian N. K. (2005). Bud microclimate and fruitfulness in *Vitis vinifera* l. Am. J. Enol. Vitic. 56, 319–329. doi: 10.5344/ajev.2005.56.4.319

[B57] Santesteban L. G. Miranda C. Urrestarazu J. Loidi M. Royo J. B. (2017). Severe trimming and enhanced competition of laterals as a tool to delay ripening in Tempranillo vineyards under semiarid conditions. OENO One 51 (2), 191–203. doi: 10.20870/oeno-one.2017.51.2.1583

[B58] Santos J. A. Fraga H. Malheiro A. C. Moutinho-Pereira J. Dinis L. T. Correia C.. (2020). A review of the potential climate change impacts and adaptation options for European viticulture. Appl. Sci. 10, 1–28. doi: 10.3390/app10093092

[B59] Shackel K. A. Ahmadi H. Biasi W. Buchner R. Goldhamer D. Gurusinghe S.. (1997). Plant water status as an index of irrigation need in deciduous fruit trees. Horttechnology 7, 23–29. doi: 10.21273/HORTTECH.7.1.23

[B60] Smith J. P. Holzapfel B. P. (2009). Cumulative responses of semillon grapevines to late season perturbation of carbohydrate reserve status. Am. J. Enol. Vitic. 60, 461–470. doi: 10.5344/ajev.2009.60.4.461

[B61] Tsubo M. Walker S. (2005). Relationships between photosynthetically active radiation and clearness index at Bloemfontein, south Africa. Theor. Appl. Climatol. 80, 17–25. doi: 10.1007/s00704-004-0080-5

[B62] Van Leeuwen C. Destrac-Irvine A. (2017). Modified grape composition under climate change conditions requires adaptations in the vineyard. Oeno One 51, 147–154. doi: 10.20870/oeno-one.2017.51.2.1647

[B63] Zapata C. Deléens E. Chaillou S. Magné C. (2004). Partitioning and mobilization of starch and n reserves in grapevine (*Vitis vinifera* L.). J. Plant Physiol. 161, 1031–1040. doi: 10.1016/j.jplph.2003.11.009 15499905

[B64] Zufferey V. Murisier F. Vivin P. Belcher S. Lorenzini F. Spring J. L.. (2012). Carbohydrate reserves in grapevine (*Vitis vinifera* L. ’Chasselas’): The influence of the leaf to fruit ratio. Vitis - J. Grapevine Res. 51, 103–110. doi: 10.5073/vitis.2012.51.103-110

