# Supplementary material for: Evaluation of carbon balance and carbohydrate reserves from forced (Vitis vinifera L.) cv. Tempranillo vines
Source: Front Plant Sci. 2022 Nov 22;13:998910. doi: 10.3389/fpls.2022.998910 (PMC9723461; doi:10.3389/fpls.2022.998910)
Supplement: Supplementary file 1 [file DataSheet_1.docx]

# Supplementary Material

**Supplementary Table 1** Trunk diameters measured in 2017 before starting the experiment of the central 16 vines of each treatment. The analysis of variance was performed at P < 0.05. ns = not significant; * = significant differences.

| **Treatment** | **Trunk Diameter (cm)** |
| --- | --- |
| Control | 38.0 ± 1.1 |
| CF_early_ | 41.2 ± 1.8 |
| CF_late_ | 40.9 ± 1.2 |
| Analysis of variance |  |
| Treatment | ns (*P* = 0.28) |
| Replicate | * (*P* <0.01) |

**Supplementary Table 2** Summary of all the vine measurements carried out in the experiment, which vines were used, when they were taken and what were the objectives of each measurement.

| **Measurements** | **Sampling** |  | **Objectives** | |  |
| --- | --- | --- | --- | --- | --- |
| **Quality and vine performance** |  |  |  | |  |
| TSS (ºBrix) | From veraison to harvest from a random sample of berries covering the four replications | | Determination of the optimal the harvest date | |  |
| TSS (ºBrix), pH and Titratable acidity (g/L) | At harvest from a sample of each replicate (n = 4) | | Determination of the grape quality at harvest | |  |
| Yield (kg/vine), Number of bunches per vine, bunch weight (kg) | At harvest from all the vines per treatment (4 vines x 4 replicates = 16 vines per treatment) | | Determination of yield and yield components of each treatment. | |  |
| Berry weight (g) | A sample of 50 berries per replicate (n = 4) at harvest | | Determination of the berry fresh and dry weight and estimating the number of berries per bunch and per vine. | |  |
| Bunch compactness (g/g) | Only in season 2019 from a sample of 10 bunches per each treatment (n = 10) at harvest. | | Assessing fruit set success | |  |
| Number of bunches per shoot | In one vine from each replication (n = 4) the number of bunches and shoots were counted before the forcing pruning (only in 2019) and at harvest (2018 and 2019) | | Assessment of carry over effects and a yield component | |  |
| Pruning weight (kg/vine) | 10 vines per treatment following the row orientation were sampled (n = 10) in winter. | | Determination of the Ravaz index and comparing vegetative growth between 2018 and 2019 | |  |
| **Vegetative growth and light interception** | **Only in 2019 using always the same four vines per treatment in which net carbon exchange was modelled (one per replicate) (n = 4)** | |  | |  |
| Biomass  (kg/vine) | At forcing dates (leaves, fruits, and shoots), at harvest (only fruits) and at the end of the season (leaves and shoots) | | Assessment of total vine biomass and biomass partitioning | |  |
| Leaf area (m^2^/vine) | From May to October every three weeks on four shoots per vine using Lopes and Pinto, 2005 method. | | Assessment of vine growth.  Input for the whole canopy photosynthesis calculation (as LAI)  Determination of LA/fruit ratio  Estimation of the leaf nocturnal respiration which is a parameter of the net carbon exchange mode  Normalization of canopy net carbon exchange by unit of leaf area (NCE/LA) | |  |
| trunk diameter (cm) | Before budburst (in March) and at the end of the season | | Assessment of the vine’s permanent structure annual growth (calculating increase of trunk cross sectional area)  Input of trunk respiration estimation (multiplied by trunk height) which is a parameter of the net carbon exchange model. | |  |
| Canopy height, width, and trunk height (m) | From March to end of the season every three weeks | | Inputs of the daily radiation interception model by Oyarzun et al., 2007 | |  |
| FIPAR (dimensionless) | From March to end of the season every three weeks from 11:00 to 12:00 GMT | | Calibrate the daily radiation interception model (dailyFIPAR) by Oyarzun et al., 2007. The modelled dailyFIPAR data was used to assess of the vine capacity to intercept solar radiation and as an input of the Charles-Edwards whole canopy photosynthesis equation. The FIPAR measurements were used to estimate the extinction coefficient (k) of the whole-canopy photosynthesis equation. | |  |
| Shoot length (cm) | From May to October every three weeks on four shoots per vine | | Estimation of the shoot dry weight used to calculate the respiration of the shoots which is a parameter of the net carbon exchange model. | |  |
| **Vine physiological measurements** | **Only in 2019 using always the same four vines per treatment in which net carbon exchange was modelled (one per replicate) (n = 4)** | |  | |  |
| Stem water potential (MPa) | From May to October on two leaves per vine (n = 8) at noon | | Assessment of vine water status | |  |
| Stomatal conductance (mol/m^2^/s) | From May to October on two leaves per vine (n = 8) at noon | | Assessment of vine water status | |  |
| Leaf photosynthesis (μmol/m^2^/s) | From May to October on two leaves per vine (n = 8) at noon | | Assessment of leaf photosynthesis  Input of the whole canopy photosynthesis Charles-Edwards equation when converted to mgCO_2_/m^2^/s | |  |
| Quantum yield (µgCO_2_/ J_PAR_) | From May to October on one leaf per vine of three replications per treatment (n = 3) from 7:00 to 11:00h GMT | | Assessment of leaf photosynthesis efficiency  Input of the whole-canopy photosynthesis Charles-Edwards equation | |  |
| **Measurements for fruit respiration calculation** |  | |  | |  |
| Weight of 20 berries (gDW) | From the pea size of berry development to harvest at intervals of 15 days. Samples were taken from 10 vines per treatment. | | Estimation of fruit dry weight used as an input to calculate fruit respiration which is a parameter of the net carbon exchange model. | |  |
| **Carbohydrate reserves** | Trunk and root samples were extracted from the same vines in each sampling date. Three vines located at the edge of the plot were used (n=3) | |  | |  |
| Trunk samples | Before budburst, at forcing dates (only forced vines) and at harvest. Two trunk samples per vine using a corer | | Trunk total non-structural carbohydrate (TNSC) analysis | |  |
| Root samples | Before budburst, at forcing dates (only forced vines) and at harvest. One sample of root (diameter between 5-10 mm) per vine was extracted using a shovel. | | Root total non-structural carbohydrate (RNSC) analysis | |  |
| **Charles-Edwards whole-canopy photosynthesis equation** | The same 4 vines in which biometric and physiological parameters were measured, were used for the model | |  | |  |
| α  (Quantum yield; µgCO_2_/ J_PAR_) | Measured as described above | | A photosynthesis efficiency parameter | |  |
| S  (Total daily integral of PAR radiation; MJ/m^2^/day) | Extracted from a nearby weather station. | | Energy received by the horizontal plane | |  |
| h  (Daylength; s) | Calculated | | The time elapsed between sunrise and sunset | |  |
| DailyFIPAR | Modelled with the Oyarzun et al., (2006) model. All the parameters are described above | | The fraction of solar radiation intercepted by the vines | |  |
| Pn  (leaf net photosynthesis; mgCO_2_/m^2^/s) | Measured as described above | | The maximum leaf photosynthetic rate | |  |
| k  (extinction coefficient) | Calculated using FIPAR measurements | | The light extinction throughout the canopy | |  |
| G  (vine spacing; m^2^) | The distance between vines in a row multiplied by the distance between two rows | | Conversion from ground area units to vine | |  |
| **Daily net carbon exchange model (NCE_m_)** | The same 4 vines in which biometric and physiological parameters were measured, were used for the model | |  | |  |
| Pn_canopy_  (gCO_2_/vine/day) | With the Charles-Edwards equation | | Calculate the net photosynthetic capacity of the canopy (only leaves) | |  |
| R_leaf_  (gCO_2_/vine/day) | With the Arrhenius equation The parameters of the equation are LA described above and respirations coefficients described in **SupplementaryTable 2** | | CO_2_ lost by the respiration of the leaves during the night | |  |
| R_shoot_  (gCO_2_/vine/day) | With the Arrhenius equation  The parameters of the equation are shoot dry weight estimated from shoot length measurements described above and respirations coefficients described in **SupplementaryTable 2** | | CO_2_ lost by the respiration of the shoots | |  |
| R_fruit_  (gCO_2_/vine/day) | With the Arrhenius equation  The parameters of the equation are fruit dry weight estimated from the weight of 20 berries measurements described above and respirations coefficients described in **SupplementaryTable 2** | | CO_2_ lost by the respiration of the fruit | |  |
| R_trunk_  (gCO_2_/vine/day) | With the Arrhenius equation  The parameters of the equation are trunk area and respirations coefficients described in **SupplementaryTable 2** | | CO_2_ lost by the respiration of the trunk | |  |
| **Daily net carbon exchange model validation** |  | |  | |  |
| Whole-canopy net carbon exchange (NCE_ch_; gCO_2_/vine/day) | From May to end of September, 14 measurements of whole-canopy net carbon exchange with open top whole-canopy chambers. | | Linear regression between then modelled NCE and measured NCE to validate and calibrate the model | |  |
|  | | | |  |  |
|  |  | |  | |  |
|  |  | |  | |  |

**Supplementary Table 3** Parameters used to calculate respiration and the reference from which they were extracted and adapted. For fruit respiration Q10 = 2 was assumed. In Escalona et al., 2012, shoot respiration was presented in nmol O_2_/gDW. We assumed a respiratory quotient = 1.

| **Organ** | **Phenology** | **Q10** | **q_m_** | **Units** |  | **Reference** |
| --- | --- | --- | --- | --- | --- | --- |
| Fruit | Bloom | 2.0 | 4.20E-04 | μmolCO2/gDW h | | Hernández-Montes et al., 2020 |
|  | Veraison | 2.0 | 2.72E-05 | μmolCO_2_/gDW h | |  |
| Shoots |  | 1.18 | 2.36 | gCO_2_/gDW h | | Escalona et al., 2012 |
| Trunk | Growing | 1.42 | 16.8 | μgCO_2_/m^2^ s | | Palliotti et al., 2005 |
|  | Not-Growing | 1.35 | 7.32 | μgCO_2_/m^2^ s | |  |
| Leaves | Bloom | 1.77 | 14.99 | μgCO_2_/m^2^ s | | Poni et al., 2006 |
|  | Veraison | 1.59 | 16.93 | μgCO_2_/m^2^ s | |  |
|  | Harvest | 1.75 | 3.38 | μgCO_2_/m^2^ s | |  |
|  | Senescence | 1.64 | 2.17 | μgCO_2_/m^2^ s | |  |

**Supplementary Table 4** Environmental conditions in and out of the whole canopy gas exchange chamber. Note that on 29^th^ May temperature and VPD in the chamber for CF_late_ were high values since a pipe obturation occurred for one hour.

| **Date** | **Treatment** | | **AvgT out (^o^C)** | **AvgT in (^o^C)** | **MaxT out (^o^C)** | **MaxT in (^o^C)** | **AvgVPD out (kPa)** | **AvgVPD in (kPa)** | **MaxVPD out (kPa)** | **MaxVPD in (kPa)** |
| --- | --- | --- | --- | --- | --- | --- | --- | --- | --- | --- |
| 29 May | Control | 20.6 | | 26.3 | 24.4 | 31.9 | 1.6 | 2.4 | 3.0 | 3.7 |
| 29 May | CF_early_ | 20.6 | | 28.5 | 24.4 | 36.0 | 1.6 | 2.9 | 3.0 | 4.6 |
| 30 May | CF_late_ | 22.1 | | 27.2 | 26.6 | 31.2 | 1.6 | 2.3 | 2.6 | 3.3 |
| 13 Jun | Control | 23.0 | | 27.8 | 26.5 | 34.3 | 1.5 | 2.3 | 2.1 | 3.8 |
| 13 Jun | CF_late_ | 23.0 | | 25.9 | 26.5 | 31.1 | 1.5 | 2.1 | 2.1 | 3.1 |
| 19 Jun | CF_late_ | 28.3 | | 31.5 | 31.1 | 35.2 | 2.6 | 3.1 | 3.2 | 4.2 |
| 31 Jul | Control | 25.0 | | 27.3 | 30.4 | 31.9 | 1.8 | 2.1 | 2.6 | 2.8 |
| 31 Jul | CF_early_ | 25.0 | | 28.3 | 30.4 | 32.1 | 1.8 | 2.1 | 2.6 | 2.8 |
| 14 Aug | Control | 25.8 | | 28.9 | 31.4 | 33.1 | 2.2 | 2.5 | 3.2 | 3.5 |
| 14 Aug | CF_late_ | 25.8 | | 30.0 | 31.4 | 35.8 | 2.2 | 3.0 | 3.2 | 4.5 |
| 5 Sep | Control | 22.7 | | 26.4 | 27.3 | 30.0 | 1.5 | 2.0 | 2.4 | 2.7 |
| 5 Sep | CF_early_ | 22.7 | | 28.3 | 27.3 | 32.1 | 1.5 | 2.4 | 2.4 | 3.4 |
| 26 Sep | Control | 23.0 | | 24.8 | 27.5 | 27.4 | 1.6 | 1.6 | 2.4 | 2.0 |
| 26 Sep | CF_late_ | 23.0 | | 25.0 | 27.5 | 28.4 | 1.6 | 1.7 | 2.4 | 2.5 |

**Supplementary Table 5** Maximum net carbon exchange per leaf area (maxNCE_ch_) and net carbon exchange (NCE_ch_), measured through whole canopy gas exchange chamber, and net carbon exchange modelled (NCE_m_).

| **Treatment** | **Date** | **maxNCE_ch_**  **(μmol m^-2^ s^-1^)** | **Daily NCE_ch_ (gCO_2_ vine^-1^ day^-1^)** | **NCE_m_**  **(gCO_2_ vine_-1_ day_-1_)** |
| --- | --- | --- | --- | --- |
| Control | 29 May | 6.33 | 64.05 | 69.67 |
| CF_early_ | 29 May | 7.72 | 58.41 | 58.53 |
| CF_late_ | 30 May | 8.31 | 51.80 | 49.30 |
| Control | 13 Jun | 7.3 | 59.20 | 51.20 |
| CF_late_ | 13 Jun | 9.54 | 70.38 | 70.37 |
| CF_late_ | 19 Jun | 12.02 | 112.55 | 105.13 |
| Control | 31 Jul | 11.86 | 105.97 | 100.48 |
| CF_early_ | 31 Jul | 11.76 | 121.67 | 120.46 |
| Control | 14 Aug | 6.2 | 119.54 | 115.55 |
| CF_late_ | 14 Aug | 12.4 | 72.20 | 81.42 |
| Control | 5 Sep | 5 | 86.35 | 86.49 |
| CF_early_ | 5 Sep | 5 | 54.65 | 63.73 |
| Control | 26 Sep | 4.5 | 63.92 | 68.01 |
| CF_late_ | 26 Sep | 8.18 | 60.68 | 56.01 |

**Supplementary Table 6** Inputs required to estimate net carbon exchange model in the experimental zone for the days in which open-top whole canopy gas exchange chambers were operating. Note that not all the measurements included a whole day data since data were adjusted for the time the vine was in the whole canopy gas exchange chamber.

| **Date** | **Treatment** | | **Phenology** | **S (MJ PAR/m_2_/day)** | **k** | **h (s)** | **α**  **(µgCO_2_/ J_PAR_)** | **maxPn**  **(mgCO_2_/m^2^/s)** | **FiPAR** | **Rfruit**  **(gCO_2_)** | **Rshoot**  **(gCO_2_)** | **Rtrunk**  **(gCO_2_)** |
| --- | --- | --- | --- | --- | --- | --- | --- | --- | --- | --- | --- | --- |
| 29 May | Control | E-L 23 | | 14.76 | 0.50 | 43200 | 3.42 | 0.44 | 0.35 | 1.82 | 0.97 | 0.34 |
| 29 May | CF_early_ |  | | 13.29 | 0.65 | 43200 | 3.42 | 0.44 | 0.29 | 0.26 | 1.14 | 0.37 |
| 30 May | CF_late_ |  | | 8.05 | 0.41 | 36000 | 3.42 | 0.44 | 0.28 | 2.57 | 0.28 | 0.30 |
| 13 Jun | Control | E-L 31 | | 10.65 | 0.43 | 28800 | 3.85 | 0.63 | 0.42 | 1.37 | 0.87 | 0.24 |
| 13 Jun | CF_late_ |  | | 7.56 | 0.55 | 32400 | 3.85 | 0.63 | 0.47 | 7.94 | 0.84 | 0.26 |
| 19 Jun | CF_late_ | E-L 32 | | 14.44 | 0.55 | 43200 | 3.91 | 0.63 | 0.46 | 0.79 | 0.72 | 0.40 |
| 31 Jul | Control | E-L 35 | | 13.12 | 0.52 | 46800 | 4.29 | 0.70 | 0.45 | 1.80 | 1.95 | 0.14 |
| 31 Jul | CF_early_ | E-L 31 | | 13.23 | 0.6 | 46800 | 4.34 | 0.73 | 0.53 | 1.13 | 3.66 | 0.15 |
| 14 Aug | Control | E-L 36 | | 12.68 | 0.6 | 43200 | 4.30 | 0.66 | 0.56 | 5.03 | 5.09 | 0.15 |
| 14 Aug | CF_late_ | E-L 31 | | 12.35 | 0.7 | 43200 | 4.49 | 0.60 | 0.37 | 1.21 | 1.69 | 0.15 |
| 5 Sep | Control | E-L 37 | | 11.24 | 0.6 | 39600 | 3.61 | 0.61 | 0.58 | 5.97 | 4.34 | 0.13 |
| 5 Sep | CF_early_ | E-L 35 | | 10.95 | 0.55 | 36000 | 3.10 | 0.65 | 0.48 | 0.39 | 2.92 | 0.13 |
| 26 Sep | Control | E-L 41 | | 9.37 | 0.44 | 39600 | 2.88 | 0.55 | 0.63 | 0.00 | 2.01 | 0.10 |
| 26 Sep | CF_late_ | E-L 35 | | 9.37 | 0.65 | 39600 | 3.14 | 0.63 | 0.48 | 0.46 | 1.66 | 0.12 |


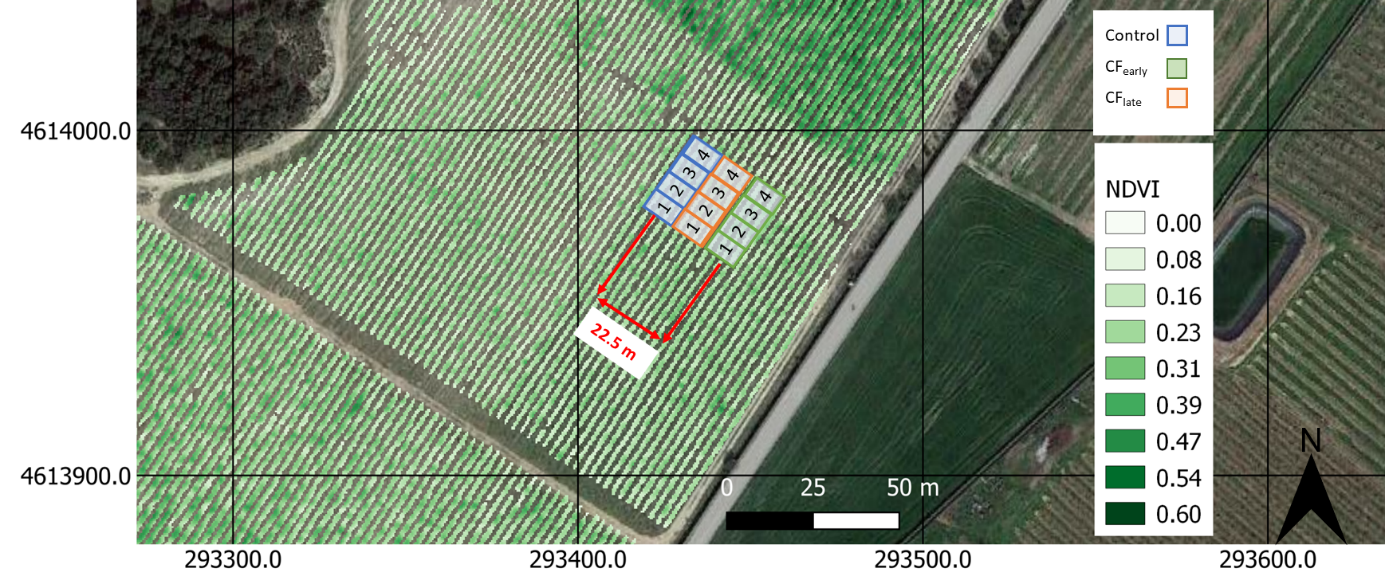


**Supplementary Figure 1** Scheme of the experimental design on an NDVI map performed in year 2016 with an airborne (all the methodology was described in Bellvert et al., 2020). The different numbers are he replicates of each treatment. The distance between vines of the same replicate of different treatments is represented in red.


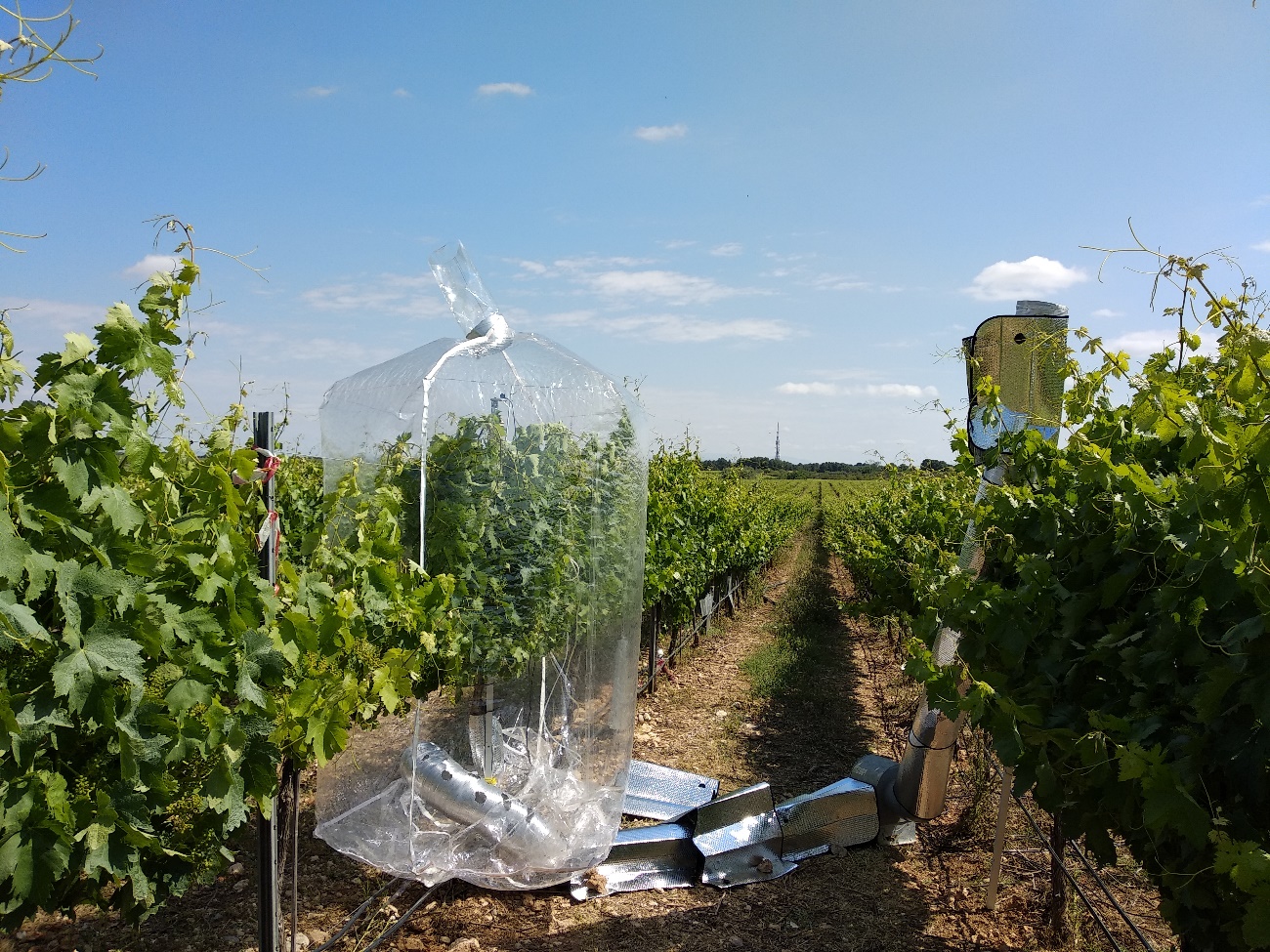


**Supplementary Figure 2** Open-top whole canopy gas exchange chamber used to validate the net carbon exchange model


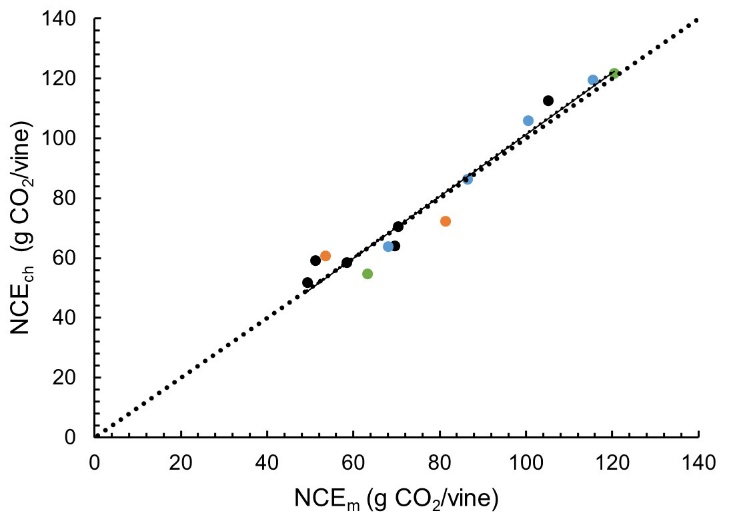


**Supplementary Figure 3** Linear regression between modelled net carbon exchange (NCE_m_) and measured using an open-top whole-canopy gas exchange chamber (NCE_ch_) (R_2_ = 0.95, $y=1.03x- 1.98$, RMSE = 5.8; NSE = 0.95). Black circles indicate measurements conducted before applying the treatments and includes vines from all three treatments in 2019. Measurements after the forcing date correspond to Control (blue), CF_early_ (green) and CF_late_ (orange). The grey dotted line means 1:1 relation.
